# Supplementary material for: Deep-LASI: deep-learning assisted, single-molecule imaging analysis of multi-color DNA origami structures
Source: Nat Commun. 2023 Oct 17;14:6564. doi: 10.1038/s41467-023-42272-9 (PMC10582187; doi:10.1038/s41467-023-42272-9)
Supplement: Supplementary file 1 — Supplementary Information [file 41467_2023_42272_MOESM1_ESM.pdf]

Supplementary Information

Deep-LASI: Deep-Learning Assisted, Single-molecule Imaging analysis of multi-color DNA Origami structures

Simon Wanninger[1], Pooyeh Asadiatouei[1], Johann Bohlen[1], Clemens-Bässem Salem[1], Philip Tinnefeld[1], Evelyn Ploetz[1,*] and Don C. Lamb[1,*]

[1.] Department of Chemistry and Center for NanoScience (CeNS)
Ludwig-Maximilians-Universität München
Butenandtstr. 5-13 81377 Munich,
Germany

[*] Corresponding authors: evelyn.ploetz@lmu.de; d.lamb@lmu.de

TABLE OF CONTENTS

Supplementary Note 1: Neural network **3**
1.1. Architecture 3
1.2. Trace classifier architecture 4
1.3. State transition classifier and number of states classifier architecture 5
Supplementary Note 2: Training **6**
2.1. Training procedure 6
2.2. Training dataset preparation 6
2.3. Simulation of single molecule traces 8
2.4. Simulation settings for training the state classifier network 12
Supplementary Note 3: Training validation **14**
3.1. Trace classifiers 14
3.2. Number-of-states classifiers 16
3.3. State-transition classifiers 16
3.4. Limitations of the state classifiers and a comparison with HMM 17
3.5. Comparison of the state classifiers with local and global HMM 19
3.6. Training and validation loss 20
3.7. Analysis of kinetic data from the kinsoft challenge 22
Supplementary Note 4: Deep-LASI versus manual analyses **23**
4.1 Comparison of Deep-LASI and manual analysis of 2-color DNA origami traces | .. 23
4.2. Influence of different training datasets and comparison to user classification 24
4.3 Deep-LASI versus manual analyses for 3-color DNA origami samples 25
4.4 Comparison of 3-color DNA origami traces selected manually and/or via DNN classification. 27
Supplementary Note 5: Manual Analysis of Single-molecule TIRF Data **28**
5.1. Work-flow 28
5.2. Camera mapping for FRET traces 29
5.3. Trace extraction and background subtraction 29
5.4. Manual trace selection and analysis 30
5.5. Accurate FRET determination 30
5.6. Hidden-Markov modeling 32
5.7. Parameters for Hidden Markov Modeling 34
5.8. Evaluation of involved FRET states and interconversion rates 34
Supplementary Note 6: Details of Deep-LASI Analyses **35**
6.1 Results for the three-color, two-state DNA origami structure with different binding site lengths 35
6.2 Kinetics of the three-color, three-state DNA origami. 36
6.3 Results for the two-color, three-state DNA origami structure 37
6.4 Kinetics as a function of Temperature 39
6.5 Analysis of previous published 2-color Hsp70 Ssc1 using Deep-LASI 39
Supplementary Note 7: DNA sequences **41**
Supplementary Note 8: Statistics Single-Molecule Data **50**
Supplementary References **51**

Supplementary Note 1: Neural network

1.1. Architecture

For the Deep-LASI software package, two different neural-network architectures are used. One architecture is for trace classification and another for the number of states and state transition classification (Supplementary Figure 1.1). Both architectures are hybrids of a convolutional neural network (CNN) and a long short-term memory (LSTM) model, which were designed using TensorFlow with Keras API.[1] The CNN framework was inspired by an omni-scale 1D-CNN, which elegantly solves the problem of finding the optimal kernel sizes by making it part of the training process.[2] Unlike traditional CNNs that operate on 2D spatial grids, the omni-scale CNN processes time series data directly by utilizing one-dimensional convolutions. These convolutional operations consider the temporal dependencies among data points, enabling the network to effectively model the sequential nature of time series data. Single-molecule Förster Resonance Energy Transfer (smFRET) data often exhibit complex patterns on different timescales, which can be crucial for accurate classification. The omni-scale CNN architecture incorporates multiple convolutional layers with different kernel sizes at the same level of depth, enabling the extraction of features at various temporal resolutions. However, the pure CNN architecture can only produce a single classification for the entire input sequence, i.e. one category per single molecule trace. The inclusion of LSTM after feature extraction of the CNN enables the sequential classification of each trace, producing a classification output at each time step. This characteristic of LSTM allows for the fine-grained detection of valid smFRET frames, photobleaching steps and state transitions. For all of our presented tasks, the omni-scale CNN LSTM hybrid architecture outperformed pure LSTM or ResNet[3] models. We did not employ the full range of prime numbers suggested for the kernel sizes as we found the accuracy did not increase above 23. Hence, the number of trainable parameters was greatly reduced. In the trace classifier model, we added a 1x1 convolution layer for dimensionality reduction to further increase efficiency without a trade-off in validation accuracy. Any down-sampling of the time dimension was avoided since the loss of this information significantly decreased the validation accuracy. This was achieved by omitting any kind of pooling or averaging layers, by zero-padding all inputs for the convolutional layers and setting the stride of all convolutional filters to 1. For kernel initialization, we used the He Normal distribution[4] as it showed the fastest convergence rate. Each convolution layer is followed by a batch normalization layer and an activation layer using the rectified linear unit (ReLU) activation function.[5]

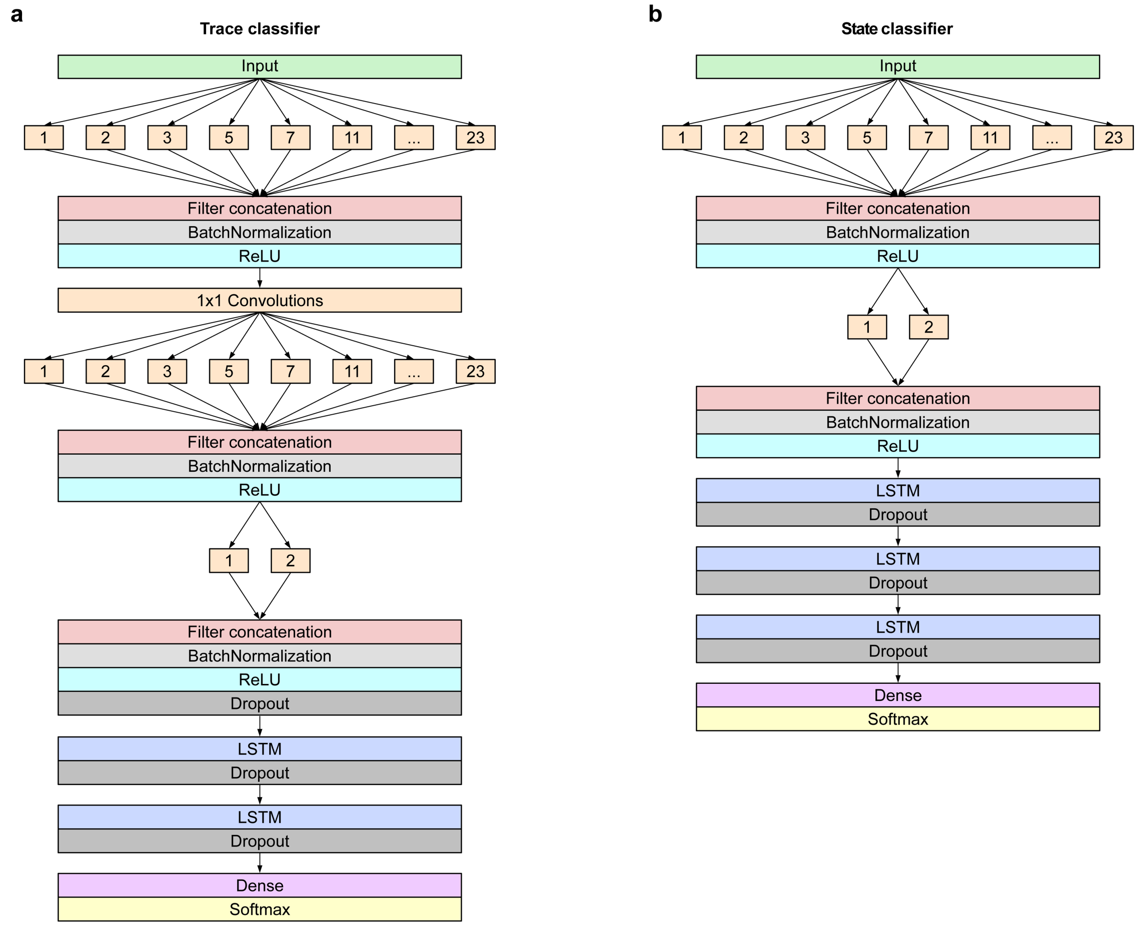

Supplementary Figure 1.1: The deep neural network architectures used for the trace classifier **(a)**, and for the state transition classifier and the number of states classifier **(b)**.

1.2. Trace classifier architecture

The trace classifier consists of four convolution layers followed by two LSTM layers and one fully connected layer as the feature extraction module (Supplementary Figure 1.1a). In the first convolution layer, the input is fed into 10 layers with 32 filters each. The kernel filter size is varied between layers with sizes given by the prime numbers from 1 to 23. All layers are stacked sequentially, i.e. they operate on the same level of depth. The second convolution layer serves as a dimensionality reduction layer with 32 filters and a kernel size of 1. The third convolution layer has the same hyperparameters as the first layer. A fourth convolution layer is added, composed of two branches with 32 filters each and kernel sizes of one and two, which allows the receptive fields of the network to cover all possible integers. The output of the CNN is fed into a LSTM layer with 128 units, followed by a second LSTM layer with 32 units and the final dense layer for classification. For the training procedure, we placed a dropout layer at a rate of 0.22 before the first LSTM layer and two dropout layers at a rate of 0.5 after the two consecutive LSTM layers to maximize the validation accuracy and reduce overfitting.

1.3. State transition classifier and number of states classifier architecture

The main difference in architecture between the transition classifier and the trace classifier are the depths and widths of the CNN and LSTM structure. The state transition classifier is composed of two convolution layers, three LSTM layers, and one final dense layer (Supplementary Figure 1.1b). The kernel sizes of the first convolution layer are prime numbers in the range of 1 to 23 with 64 filters each. The second convolution layer has kernel sizes of one and two with 32 filters each. The CNN substructure is directly followed by three LSTM layers with 128 units. Dropout layers are placed after each LSTM layer using a rate of 0.5. At the end, a fully connected layer (or dense layer) is used to reduce the output of the network into the number of given categories.

SUPPLEMENTARY NOTE 2: TRAINING

Although an optimized architecture is important and improves the efficiency of a neural network, the functionality of the network rises and falls with the dataset used for training the network. There are a number of important factors to consider when training a neural network. Typical pitfalls include using a dataset that is too small in size or contains an intrinsic bias, or overfitting the training data. A neural network is biased towards features it has seen before. Hence, the training dataset should include the various possibilities (e.g. number of FRET states, kinetic rates, signal-to-noise ratios). If the training dataset includes any bias, this will also be reflected in the output of the algorithm. One way that bias can be introduced into the training dataset is from unbalanced sampling of categories. For example, for the trace classifier models, it is important that the training dataset includes the same number of traces from each category. It is also important to know when to stop the training process. Neural networks can be overtrained, meaning that they memorize the training data but do not learn the general principles behind it. Below we discuss the details of the training procedure and how we optimized the training process.

2.1. Training procedure

Both the trace classifier and transition classifier models were trained using the Adam optimizer with the default settings[6]. We used a hybrid method of increasing the batch size and lowering the learning rate during training. The entire training set of ca 200,000 traces is feed into the neural network in batch sizes of 32 traces until the network has seen all traces (referred to as an epoch). After the network has seen all traces once, some input units are randomly set to 0 using dropout layers and the dataset is fed again in batches to the neural network in the next epoch. The dropout layers reduce overfitting and allow generalization of the learned information. When the validation loss is not significantly lowered within 4 epochs, the batch size is doubled. An initial learning rate of 0.001 was decreased analogously by factors of 10 after a batch size of 512 was reached.

2.2. Training dataset preparation

To generate training datasets, we found the approach of using simulations, originally described in Thomsen et al.[7], to be the most promising. This is especially true for three-color models capable of detecting state transitions or photobleaching events of each dye individually. A manual collection of labeled traces on a scale large enough for adequate training would be prone to biases and/or errors due to incorrect trace identification. In addition, the datasets would not be optimized for microscope setups with different characteristics. The signal and noise characteristics of smFRET data is well enough understood that simulated data can accurately reproduce the characteristics of real data. Beside the architecture itself, the main differences between our trace classifiers and the DeepFRET model[7] is the ability to classify one-color and three-color data and to predict the photobleached frames of each fluorophore separately. We adopted the categories 'dynamic', 'static', 'noisy', and 'aggregate' while implementing

additional categories for all possible photobleaching events. The ‘artifact’ category includes false localizations, overestimated background and random perturbations of the intensity traces. For the simulated data, idealized intensity traces for each primary category (i.e. non-photobleaching category, 'dynamic', 'static', 'noisy', 'aggregate' and 'artifact') are generated and then photobleaching steps are added for the different dyes by randomly determining the survival time of the dye from a given exponential distribution. The addition of photobleaching as well as other processes in the simulation may lead to alterations in the label of the given trace. For example, a simulated 'dynamic' trace that does not undergo a transition before photobleaching or by the end of the trace would be recategorized as 'static'. To ensure that the network sees the same number of frames for the different categories including all the photobleached categories, the number of traces selected for the training set needs to be balanced. Hence, we begin by simulating ~250,000 traces of 500 frames for each primary category. After including photobleaching, the number of labeled frames for each category is determined. The category with the minimum number of frames determines a threshold at which additional traces, depending on their present classification, are added or excluded from the final training dataset. The typical number of traces included in the final training dataset is approximately 200,000. This balancing procedure ensures that no category is over- or underrepresented across all frames and minimizes biases of the trained deep neural networks. Supplementary Figure 2.1 shows the cumulative distribution of category labels in each training set used for trace classification.

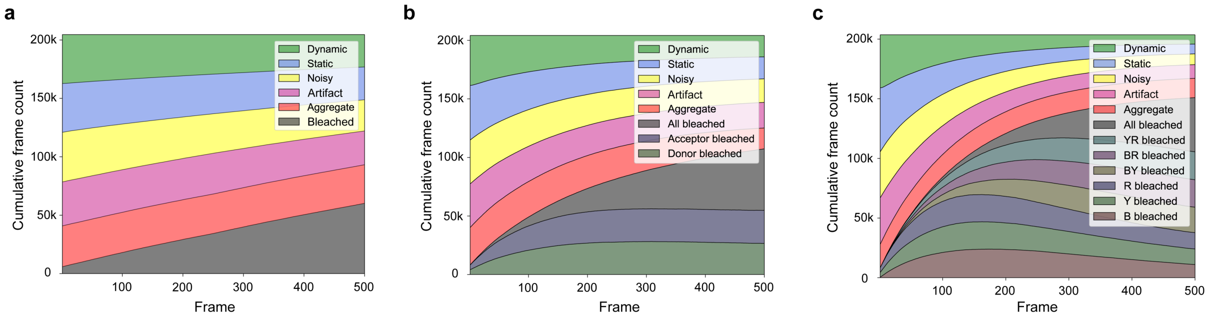

Supplementary Figure 2.1: Cumulative distribution of labeled categories in training datasets for one-color **(a)**, two-color **(b)** and three-color data **(c)**. The same dataset was used for training the continuous wave two-color network as for the two-color ALEX network with the exception that the ALEX channel was not included.

For training of the state classifiers, only frames where all dyes are photoactive are included and hence photobleaching can be ignored for training these classifiers. The number of categories in the training sets then equals the number of states in the model. The visible states are first counted and sorted according to their chronological order before the state of each frame is assigned. This results in the first observed state always receiving the first label regardless of the FRET efficiency value and hence a state label only corresponds to a particular FRET value when a given dataset is analyzed globally.

2.3. Simulation of single molecule traces

Single-molecule intensities traces are simulated by first initializing the number of traces to be simulated (250,000 in our case) and the probability of the trace being a single-molecule trajectory or an 'aggregate'. All parameters used for simulating single-molecule traces are given in Supplementary Table 2.1. An idealized FRET efficiency trajectory is then generated using an HMM routine[8] in one, two or three colors where the FRET efficiency (or efficiencies) is randomly selected between 0.01 and 0.99. For single molecule trajectories, the number of states in the trajectory is selected with a probability of containing only a single state (45 % in our case for 'static' traces) and the remaining probability (55 % here) is equally distributed between two, three and four states ('dynamic'). In the case of 'dynamic' traces, the FRET efficiency or efficiencies of each state are randomly selected from a uniform distribution between 0.01 and 0.99. The difference in FRET efficiency for the different states has to be above a given threshold (0.1 in our cases). If this is not the case, new FRET efficiencies are randomly selected until this criterion is fulfilled. The transition rates are generated by taking the inverse of the dwell time to exposure ratio drawn from a uniform distribution between 1 and 100. The generated transition matrices are then, in general, non-symmetric. Hence, we use the transition rate matrix to calculate the probability of which state is observed first. We do this by using the least-squares solution to the matrix equation $Ax = b$, where A is the transition matrix and x is the probabilities for observing the different states. While the calculation of the state equilibrium is not mandatory for the classification accuracy, it ensures that the output matches the ground truth input of a defined transition matrix, which was used for benchmarking the transition classifier. Once the initial state has been selected, the parameters are fed into an HMM routine[8] and a state trajectory is generated. 'Aggregate' traces are always assumed to be static (uncorrelated dynamics are categorized as 'artifact'), but the number of dye-pairs is generated from a Poisson distribution with randomly selected FRET efficiencies between each pair. Next, the idealized FRET efficiency trace is converted into normalized fluorescence intensity traces for the donor and acceptor molecules based on the FRET efficiency (discussed in more detail below). Next, photobleaching of the fluorophores are included into the trajectories. The frame at which each fluorophore photobleaches is randomly drawn from an exponential distribution. Upon photobleaching, the affected channel intensities are either set to 0 for both channels for donor photobleaching, 1 for the donor intensity upon acceptor photobleaching or recalculated using the two-color FRET equations (for three-color simulations). Blinking is then added to a fraction of the traces where each dye has a probability of being in a short-lived dark state (Supplementary Table 2.1). At this point, 'artifact' traces are generated from 'static' or 'dynamic' traces with a given probability by subtracting a constant from the trace (to simulate overestimation of the background correction), adding random fluctuations to the total intensity (to simulate among other things new molecules or aggregates flowing through the observation volume or simulating molecules in the background mask), flipping the traces (to simulate molecules that turn on during the experiment) and/or adding non-correlated signal in the different channels. To account for non-uniform brightness of the individual molecules, all excitation channels are multiplied by a scaling factor that is randomly selected from uniform distributions. In particular, the red channel after red excitation, I_{RR}, can reach scaling factors

up to three times higher than the other two channels. This allows the trace classifier to correctly analyze datasets in which high red laser powers were used to increase statistics for the calculation of correction factors and for making sure only a single red fluorophore is present. Without intensity scaling, the trace classifier strongly favors the aggregate category for traces with imperfect stoichiometry even when no second bleach step is present. With a given probability, additional small fluctuations in the total intensity are also added to the traces to simulate experimentally observed system instabilities (assuming sinusoidal oscillations of randomly determined frequency and amplitude). Next, we incorporate spectral crosstalk, direct excitation and differences in detection efficiency into the data by randomly selecting the respective parameters from a uniform distribution (see Supplementary Table 2.1). In the last step, we add two or three types of random noise to the traces. The first component is intensity-independent background noise drawn from a Poisson distribution. The second component considers intensity-dependent noise contributions (i.e. shot-noise) by drawing values from Gaussian distributions. There are different descriptions of how to treat noise from EMCCD cameras. According to Basden et al., the variance in shot noise due to the EM gain is increased by a factor of two.[9] This corresponds to a rescaling of the Gaussian distribution mentioned above. Hirsch et al describe the additional noise from EM-CCD cameras using a gamma distribution.[10] Hence, with a given probability, we also add a third component to the noise modeled using a gamma distribution with random amplitude. After adding noise to the trajectories, we then recategorize traces with high noise as 'noisy'. This is done by recalculating the FRET efficiency trace or traces from the intensity data. When the standard deviation for static traces or individual states of a dynamic trace are above the given threshold (Supplementary Table 2.1, we used 0.25), the trace is categorized as 'noisy'. Finally, the classification of the individual traces is checked and, if necessary (for example a dynamic trace that photobleaches before a transition is observed), recategorized. The dataset is then balanced, as discussed above, each trace normalized to its maximum value and then used for training.

For one-color traces, we simulated the intensity of the donor molecule although, for a single channel, it does not make a difference. The donor intensity (we refer to it as *YY* here) is given by:

$$I_{YY} = 1 - E_{YR} \qquad \textbf{Eq. 2.1}$$

Since only one dye and one channel is observed, there is only one photobleaching category and no correction factors are included. The photoactive state of the acceptor molecule is still calculated and its influence on the donor intensity incorporated into the trace. For calculation of 'aggregates', fluorescent dye-pairs are added to the trace but only the donor signal is considered. Furthermore, the amount of noise is not quantified by the standard deviation of the FRET efficiency but by the signal-to-noise ratio of the channel intensity, which is defined as:

$$\mathrm{SNR} = \frac{\mu}{\sigma} \qquad \textbf{Eq. 2.2}$$

where μ is the mean signal intensity of the observed state and σ is its standard deviation. When the signal-to-noise ratio falls below the given threshold, the trace is classified as 'noisy'.

For two-color FRET simulations, we use the same approach as described in the following section for 3-color FRET but we only consider the equations necessary for 2-color FRET, i.e. all equations including the yellow/red FRET pair.

For generating three-color FRET data, the distances and Forster radii between all three fluorophores need to be considered as they are interrelated. Assuming a minimum FRET efficiency of 0.01, the generated FRET states of the first two randomly drawn FRET pairs put constrains on the maximum possible distance for the third FRET pair. To guarantee a uniform distribution of possible FRET combinations, we randomly select two of the three FRET pairs and their corresponding FRET efficiencies. Using the two selected FRET efficiencies, a lower limited is calculated for the third FRET pair, which depends on the Förster radii of the first two FRET pairs. For example, when the yellow-red dye-pair is generated last, the dye-dye separation for r_{BY} and r_{BR} are calculated and then used to determine the minimum FRET efficiency (i.e. maximum separation for the third dye-pair) as given below:

$$r_{BY} = R_{0,BY}\left(\frac{1}{E_{BY}} - 1\right)^{\frac{1}{6}} \qquad \textbf{Eq. 2.3}$$

$$r_{BR} = R_{0,BR}\left(\frac{1}{E_{BR}} - 1\right)^{\frac{1}{6}} \qquad \textbf{Eq. 2.4}$$

$$E_{YR,min} = \frac{1 - 0.01}{1 + \left(\frac{r_{BY} + r_{BR}}{R_{0,YR}}\right)^{6}} + 0.01 \qquad \textbf{Eq. 2.5}$$

where the Forster radii $R_{0,BY}$, $R_{0,BR}$ and $R_{0,YR}$ are sampled over values that are typically available using commercially available dyes pairs. The FRET efficiency $E_{YR,min}$ represents the lower boundary used to randomly scale the FRET trace of the yellow-red FRET pair in a correlated or anti-correlated manner. When a different dye-pair is generated last, the same equations are used where the indices are changed accordingly. For dynamic traces, this procedure is performed for all states. Once we have selected the FRET efficiencies for the different dye-pairs and states, we then convert them what would be observed for a two-color experiment. The YR dye-pair is already a two-color FRET efficiency and does not need to be corrected. When all three fluorophores are photoactive, the blue dye may be quenched by two acceptors. In this case, the distance-related FRET efficiencies E_{BY} and E_{BR} need to be converted into the apparent FRET efficiencies $E_{BY,app}$ and $E_{BR,app}$ via:

$$E_{BY,app} = \frac{E_{BY}(1 - E_{BR})}{1 - E_{BY}E_{BR}} \qquad \textbf{Eq. 2.6}$$

$$E_{BR,app} = \frac{E_{BR}(1 - E_{BY})}{1 - E_{BY}E_{BR}} \qquad \textbf{Eq. 2.7}$$

Since the input data for the neural networks are normalized, the channel intensities are initialized as follows:

$$I_{BB} = 1 - E_{BY,\mathrm{app}} - E_{BR,\mathrm{app}} \quad \textbf{Eq. 2.8}$$

$$I_{BY} = E_{BY,\mathrm{app}}(1 - E_{YR}) \quad \textbf{Eq. 2.9}$$

$$I_{BR} = E_{BR,\mathrm{app}} + E_{BY,\mathrm{app}}E_{YR} \quad \textbf{Eq. 2.10}$$

$$I_{YY} = 1 - E_{YR} \quad \textbf{Eq. 2.11}$$

$$I_{YR} = E_{YR} \quad \textbf{Eq. 2.12}$$

$$I_{RR} = 1 \quad \textbf{Eq. 2.13}$$

Upon photobleaching of one of the dyes in the three-color experiments, the system then reverts into the two-color case:

$$I_{BY,2c} = -\frac{I_{BB}E_{BY}}{E_{BY} - 1} \quad \textbf{Eq. 2.14}$$

$$I_{BR,2c} = -\frac{I_{BB}E_{BR}}{E_{BR} - 1} \quad \textbf{Eq. 2.15}$$

I_{BB} is still determined by using

$$I_{BB,2c} = 1 - E_{BY} \quad \textbf{Eq. 2.16}$$

$$I_{BB,2c} = 1 - E_{BR} \quad \textbf{Eq. 2.17}$$

where FRET to the blinking fluorophore is set equal to zero in two-color sections of the trace. Blinking events are treated the same way as photobleaching during the frames where the one dye is off and the channel intensities are either set to 0 or recalculated using Eq. 2.16/2.17.
In three-color experiments, each channel has its own set of correction factors for differences in detection efficiency and quantum yield, γ, direct excitation, de, and spectral crosstalk, ct. The values are randomly drawn from a wide uniform range and implemented in the following order. First, the FRET channels are multiplied by the corresponding γ-factor:

$$I_{BY} = \gamma_{BY}I_{BY} \quad \textbf{Eq. 2.18}$$

$$I_{BR} = \gamma_{BR}I_{BR} \quad \textbf{Eq. 2.19}$$

$$I_{YR} = \gamma_{YR}I_{YR} \quad \textbf{Eq. 2.20}$$

The crosstalk of the blue fluorophore leaking into the yellow and red channel after blue excitation are given by:

$$I_{BY}^{ct} = \frac{ct_{BY}}{1 + (ct_{BY} + ct_{BR})} I_{BB} \qquad \textbf{Eq. 2.21}$$

$$I_{BR}^{ct} = \frac{ct_{BR}}{1 + (ct_{BY} + ct_{BR})} I_{BB} \qquad \textbf{Eq. 2.22}$$

where ct_{BY} and ct_{BR} denote the randomly drawn crosstalk factors (Supplementary Table 2.1). Spectral crosstalk of the yellow fluorophore into the red channel is calculated using:

$$I_{(BY)R}^{ct} = \frac{ct_{YR} I_{BY}}{(1 + ct_{YR})} \qquad \textbf{Eq. 2.23}$$

$$I_{YR}^{ct} = \frac{ct_{YR} I_{YY}}{(1 + ct_{YR})} \qquad \textbf{Eq. 2.24}$$

The observed intensities including all correction factors are determined by:

$$I_{BB,obs} = I_{BB} \qquad \textbf{Eq. 2.25}$$

$$I_{BY,obs} = \gamma_{BY} I_{BY} + de_{BY} I_{YY} - I_{(BY)R}^{ct} \qquad \textbf{Eq. 2.26}$$

$$I_{BR,obs} = \gamma_{BR} I_{BR} + de_{BR} I_{RR} + E_{YR} \frac{de_{YR} I_{YY}}{(1 - E_{YR})} \qquad \textbf{Eq. 2.27}$$

$$I_{YY,obs} = I_{YY} - I_{YR}^{ct} \qquad \textbf{Eq. 2.28}$$

$$I_{YR,obs} = \gamma_{BY} I_{YR} + de_{YR} I_{RR} \qquad \textbf{Eq. 2.29}$$

where de_{BY}, de_{BR} and de_{YR} are the randomly drawn direct excitation factors from a uniform distribution (Supplementary Table 2.1).

While the non-smFRET categories ‘noisy’ and ‘aggregate’ mimic experimental data, the category ‘artifact’ is primarily designed to increase the robustness of the trace classifier. It is important to note that the accuracy of a trained neural network to distinguish between an ‘artifact’ and any other category depends on the number of traces which are labeled as ‘artifact’ but maintain a strong resemblance to the original trace. For the goal of increasing robustness, it is therefore not desirable to achieve 100% prediction accuracy as it would be caused by too easily identifiable perturbations in the training dataset.

2.4. Simulation settings for training the state classifier network

Sixteen pre-trained deep neural networks are provided for state classification. Four models account for the classification and segmentation of time trajectories obtained from measurements using single-channel data acquisition, two-color FRET with continuous wave excitation, two-color FRET with ALEX, and three-color FRET with ALEX. For each type of experiment (one, two and three-color), we provide three state-transition-classifiers trained on either two, three or four observed states. The state classifier networks only use traces as input

that are categorized as dynamic. Hence, the training datasets only contain valid FRET traces with at least one transition, a minimum state difference of 0.1 in FRET efficiency and no photobleaching. The transition rates are generated by drawing random dwell time to exposure ratios between 1 and 100 from a uniform distribution. Traces with a state-wise FRET distribution width above 0.25 on average are excluded from the training dataset. After a dynamic trace is simulated, it is labeled according to state occupancy. Here, the first observed state always receives the first label regardless of the FRET efficiency, followed by the next observed states until the maximum number of states is reached. For three-color FRET data, every transition regardless of the dye is treated as a new state. For a two-state model, transitions of one dye can be described whereas the multi-state model also considers transitions of two dyes. Thus, we have trained the network to recognize four different states. For a system with three independently moving dyes, a minimum of 9 states would be possible in one trace. Expert users can generate a corresponding training dataset by setting the algorithm parameter 'static dyes' to 'None'.

Supplementary Table 2.1. Simulation parameters for the training datasets.

Number of Frames	Maximum number of states	Minimum state difference	Dwell time / exposure ratio	Artifact probability	Stoichiometry tolerance
500	4	0.1	1-100	0.25	0.1-0.9
Aggregate probability	Blue intensity scaling	Yellow intensity scaling	Red intensity scaling	Mean bleaching frame	Blinking probability
0.1	0.7-1.3	0.7-1.3	0.5-2	400	0.2
Gamma blue/yellow	Gamma blue/red	Gamma yellow/red	Direct excitation blue/yellow	Direct excitation blue/red	Direct excitation yellow/red
0.7-2	0.49-2.6	0.7-1.3	0-0.4	0-0.2	0-0.3
Crosstalk blue/yellow	Crosstalk blue/red	Crosstalk yellow/red	Noise scaling factor	Gamma noise prob.	Noisy threshold
0-0.6	0-0.2	0-0.3	0-0.9	0.8	0.25 (SNR: 1.5)

SUPPLEMENTARY NOTE 3: TRAINING VALIDATION

In the following sections (3.1–3.3), the final validation of every deep neural network is shown via confusion matrices. Approximately 20,000 new simulated traces were generated and fed into each trained model. Each row of the confusion matrices represents the instances in a ground truth category while each column represents the instances in a predicted category. The diagonal values report the percentage of true positives and true negatives whereas the off-diagonal values are the false negatives and false positives.

3.1. Trace classifiers

Confusion matrices for the trace-classifier networks are shown in Supplementary Figures 3.1 and 3.2. The single-channel classifier has the lowest overall performance, in particular, due to a higher rate of falsely classifying random perturbations in ‘artifact’ frames (88 % precision) and misinterpreting ‘dynamic’ traces as ‘static’ (5 % false negative rate). The two-color and three-color models achieve similar accuracies for recovering smFRET frames with at least 93 % precision in correctly predicting ‘dynamic’ frames and 96 % precision for ‘static’ frames. In general, most of the false predictions concerning smFRET categories come from the high resemblance of ‘static’ frames, ‘dynamic’ frames with low contrast between states and ‘noisy’ frames close to the defined threshold. Here, the tolerance towards noise, defined as the mean standard deviation of the observed FRET efficiencies for all states, was set to 0.25. The highest sensitivity for detecting photobleached dyes (>98 %) is achieved by ALEX-enabled models for two- and three-color data. The continuous wave models depend on the contrast in intensity between the quenched and photobleached dyes, causing a significant decrease in sensitivity down to 91 % for detecting a photobleached acceptor. However, falsely predicted ‘acceptor bleached’ frames were mostly misclassified as either ‘aggregate’ or ‘artifact’ and would still be excluded from further analysis.

In addition to the confusion matrix for all available categories, we also calculated a binary trace classifier confusion matrix where we separated the frames into those that were accepted for further analysis (i.e. from ‘static’ and ‘dynamic’ traces without photobleaching) and those that were rejected (‘photobleached’, ‘aggregate’, ‘artifact’ and ‘noisy’ traces and/or frames). All trace classifier models achieve a minimum combined precision of 97 % in predicting smFRET categories, i.e. ‘static’ or ‘dynamic’, and 96 % in predicting non-smFRET categories (Supplementary Figures 3.1 and 3.2).

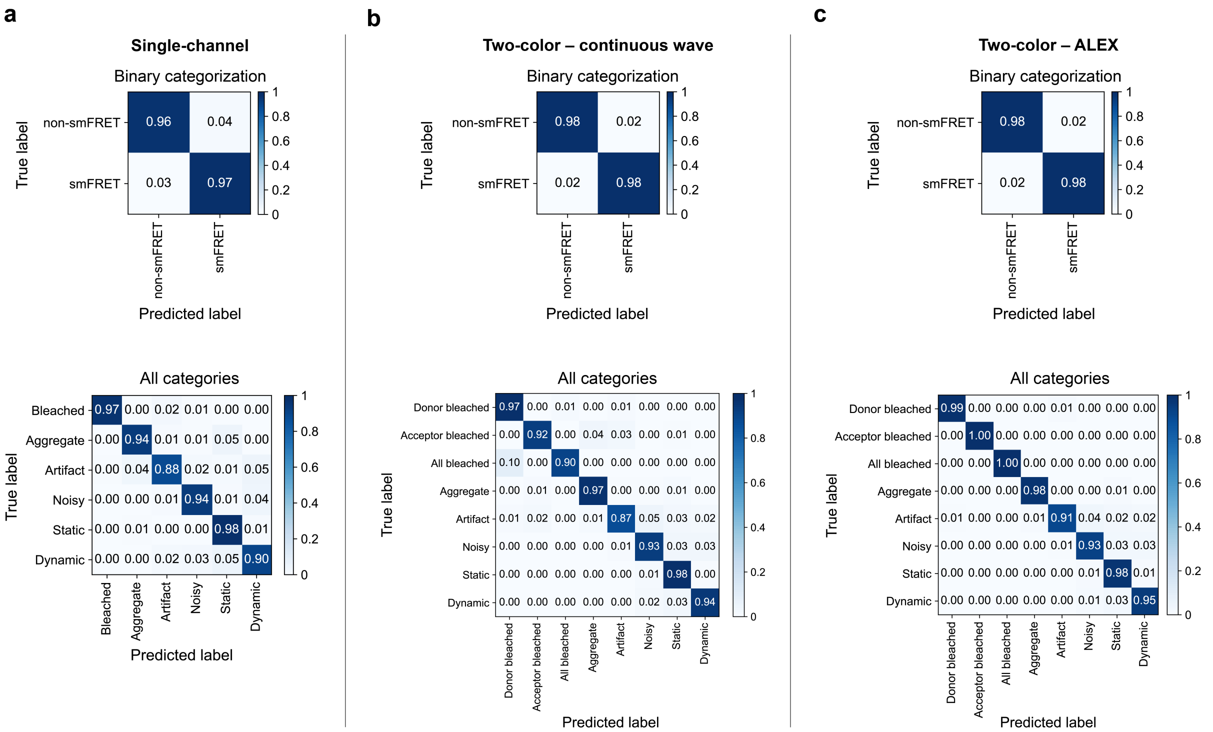

Supplementary Figure 3.1: Confusion matrices for 1-color and 2-color trace classification. Prediction accuracies depicted as confusion matrices for the **(a)** single-channel, **(b)** two-color continuous wave and **(c)** two-color ALEX models. The upper panels show the binary assignments into valid smFRET and non-smFRET categories. The detailed categorization is shown in the lower panels.

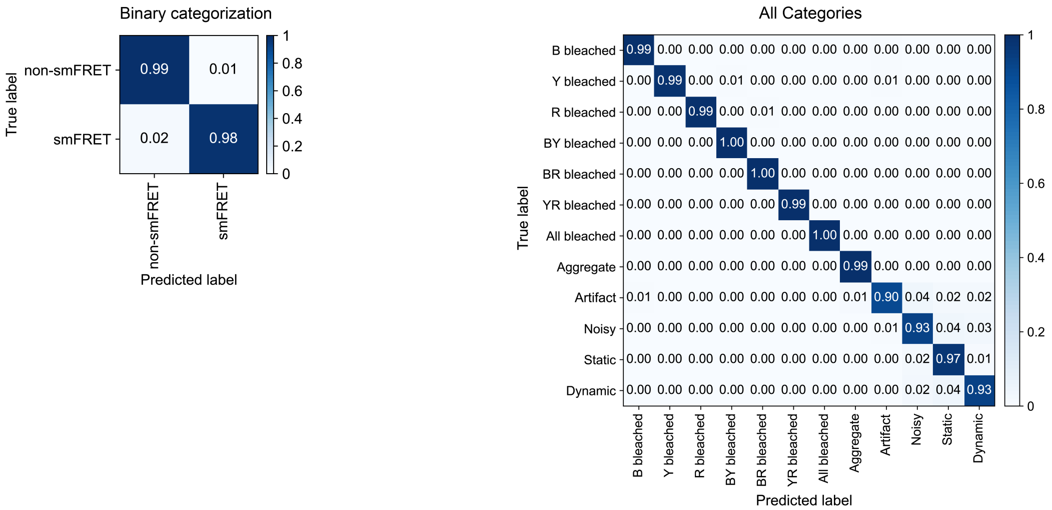

Supplementary Figure 3.2: Confusion matrices for 3-color trace classification. The left panel shows the binary assignments into valid smFRET and non-smFRET categories. The detailed categorization is shown in the right panel.

3.2. Number-of-states classifiers

After classifying the individual traces, the dynamics are analyzed. One option is to classify the number of states in a particular trace, i.e. to run the number of states classifier for the type of data measured. Supplementary Figure 3.3 shows validation of the deep neural networks trained on traces containing the given number of observed states. Only traces classified as ‘dynamic’ by the trace classifiers serve as input, hence the first category is for two observed states. The category of five observed states serves as a safeguard against traces that may be out of the scope of the pretrained state transition classifiers. All models achieve a high accuracy of at least 98 % in distinguishing two-state from multi state traces. The lowest accuracies are achieved in separating four-state from five-state traces, ranging from 86 % (single-channel) to 89 % (5-channels). The overall performance increases with increased number of available channels. As only dynamic information is considered in the state classifiers, the presence of the ALEX channel, though very useful for the trace classification, is no longer relevant.

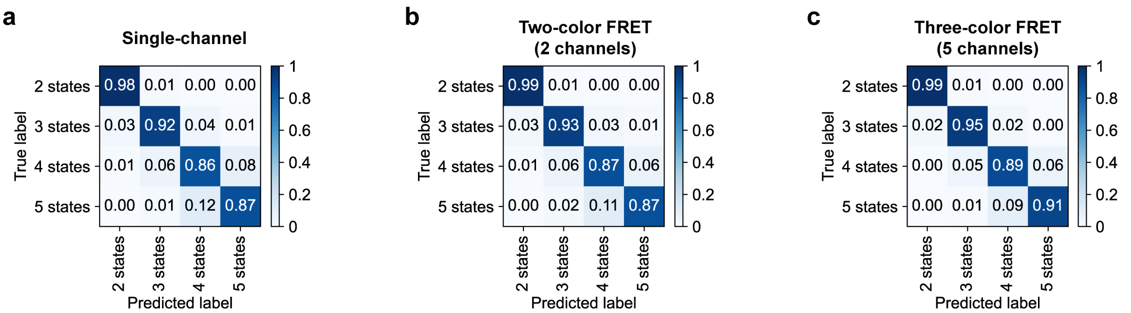

Supplementary Figure 3.3: Confusion matrices for number of states classification. Confusion matrices for the **(a)** single-channel, **(b)** two-color FRET and **(c)** three-color number of states classifiers.

3.3. State-transition classifiers

After estimating the number of states in a dataset, the state trajectories of the individual dynamic traces are determined. This section summarizes the validation of the deep neural networks trained on the state occupancy and therefore also on the state transitions (Supplementary Figure 3.4). The performance does not differ significantly for the two-state models with a minimum of 97 % precision for predicting the correct state and a minimum of 84 % precision for four-state systems. The performance for multi-state models increases when more channels are available

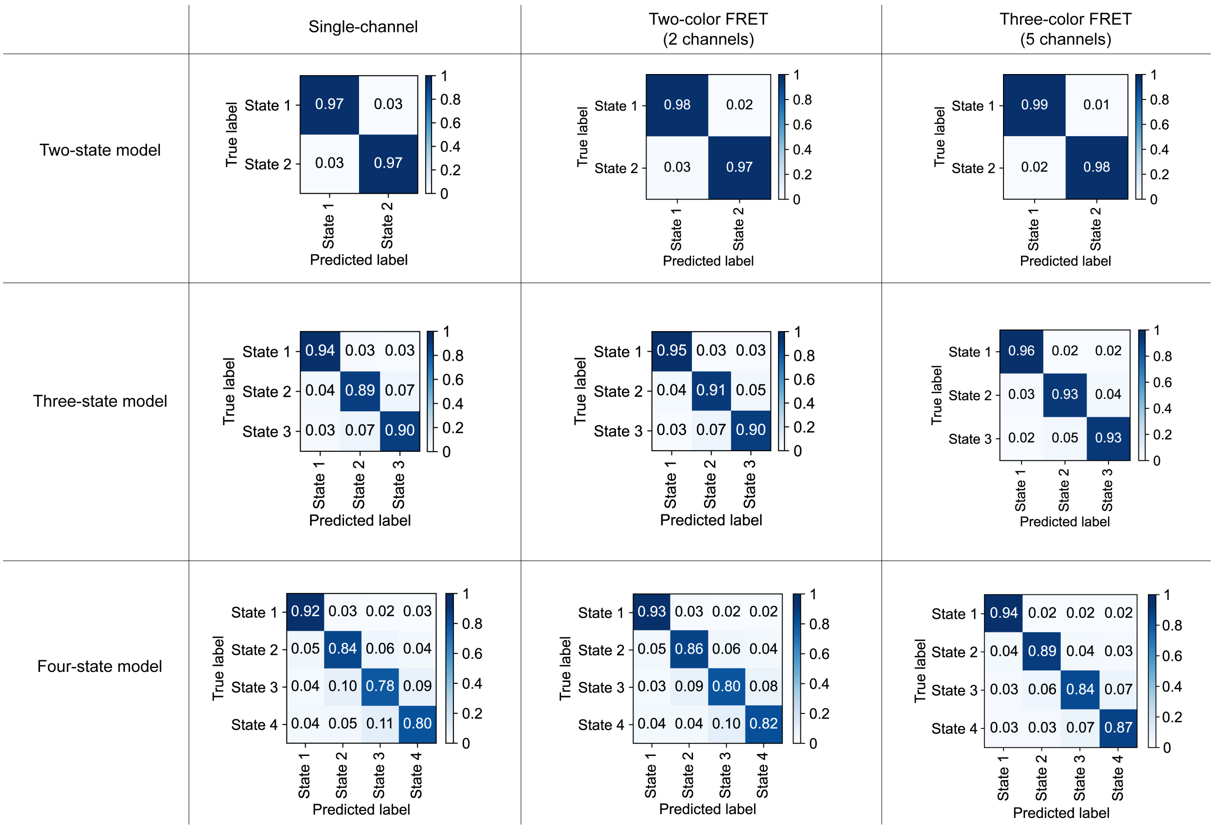

Supplementary Figure 3.4: Confusion matrices for state classification. Confusion matrices for the single-channel (first column), two-color FRET (second column) and three-color state classifiers (third column) and their corresponding two-state (first row), three-state (second row) and four-state models (third row).

3.4. Limitations of the state classifiers and a comparison with HMM

This section provides additional benchmarks and a comparison of the results from the state classifiers with HMM. First, we investigated how the performance of HMM and our state classifiers depends on noise (i.e. the width of the FRET distribution), difference between FRET states, the kinetic rates (dwell time to exposure ratio) and the length of observation time (number of frames) for dynamic transitions between two FRET states. For the three-color simulations, only the FRET distribution width of the yellow-red dye pair was used as the ground truth parameter to keep the continuity with two-color FRET traces and avoid averaging inconsistencies. Supplementary Figure 3.5 shows interpolated maps of the precision of state label recovery for all models and were generated using approximately 300,000 simulated traces for each condition. The precision is the fraction of true positives divided by the sum of true positives and false positives for the state label predictions. Each map shows the precision dependency on the amount of signal noise with two of three parameters being fixed, namely the FRET state contrast (0.2), the transition rate (0.05/frame) and the number of frames (500). In general, at a fixed transition probability and number of frames (top row), the precision decreases with broader FRET distributions and smaller differences between the FRET states. All models are able to achieve a precision of at least 90% for FRET differences above 0.2 and FRET distribution widths below 0.10 with the state classifiers outperforming HMM only at high noise

levels above 0.25. For a fixed contrast between FRET states (0.2) and total number of frames (500) (Supplementary Figure 3.5, middle row), the precision of HMM remains largely independent of the dwell time to exposure ratio at a constant noise level. All DNN state classifiers show a similar overall performance but achieves a higher precision than HMM at higher noise levels for larger dwell time to exposure ratios. For fixed FRET states and kinetic rates (Supplementary Figure 3.5, bottom row), trace length has little influence on the precision of all models below ~100 frames and the precision slightly increases for all models/classifiers above 100 frames. Again, the DNN outperform HMM at higher noise levels. In summary, while the precision does not differ significantly between the single-channel and two-channel state classifiers, the five-channel model used for three-color FRET shows an increased performance of up to ~10 % at high noise levels. Due to the five available channels, the signal-to-noise ratio is effectively increased which leads to higher precision and accuracy as soon as the signal noise becomes a limiting factor for the other models. In addition, DNN models still predict transitions in high noise trajectories, however with decreased confidence, whereas HMM no longer finds transitions at high noise.

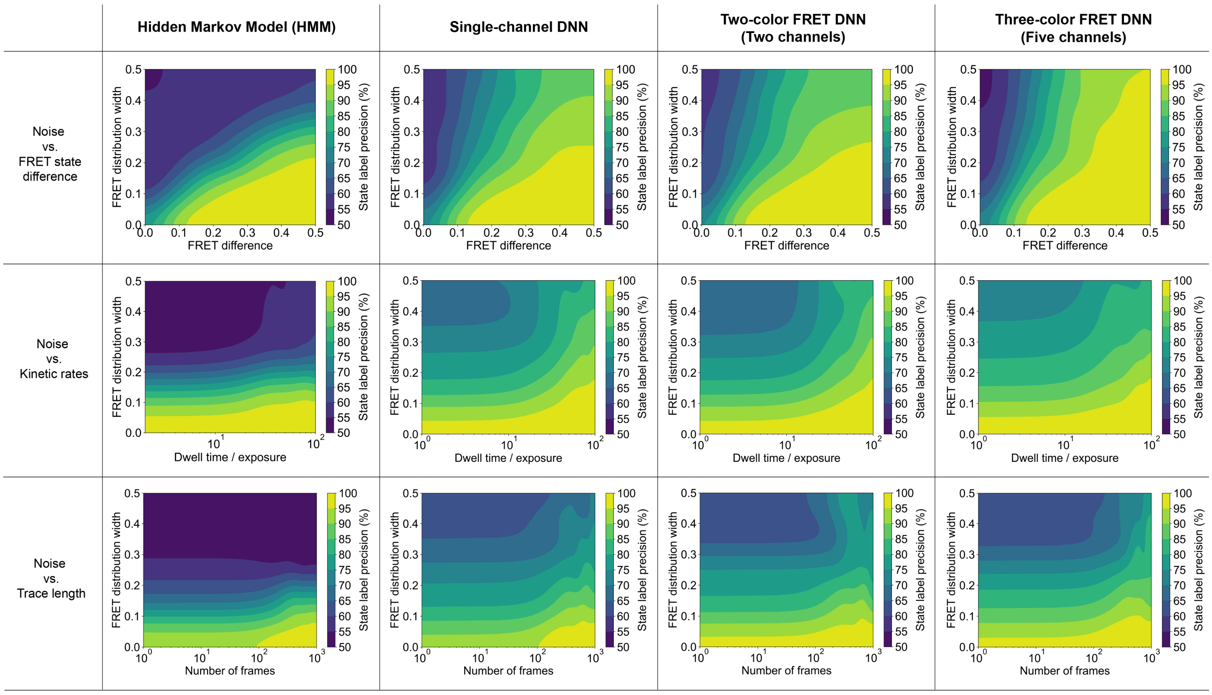

Supplementary Figure 3.5: Deep-LASI state prediction compared to HMM. Precision of the state-label recovery for HMM and for the state transition classifiers as a function of noise (i.e. width of the FRET distribution), contrast between FRET states and the kinetic rates (dwell time to exposure ratio). Each map shows the precision dependency on the noise and one additional parameter: the contrast between FRET states (top row), the kinetic rate (middle row) and the number of frames (bottom row). One dataset with ~300,000 traces was generated for each row with the corresponding two of the three parameters fixed (FRET efficiency for yellow/red: 0.4 and 0.6, transition probability: 0.05/frame, and number of frames: 500). The noise is defined as the mean standard deviation of the FRET signal from both states. The lower limit of the precision is set to 50 % since it represents the highest amount of uncertainty for two states.

3.5. Comparison of the state classifiers with local and global HMM

We also compared the performance of Deep-LASI with a local and global HMM analysis (Supplementary Figure 3.6). In a global HMM analysis, the algorithm can learn from a large dataset rather than being limited to the number of frames in a single trace. For the comparison, we simulated 2000 traces of 500 frames (1 million data points) of three-color data having two states. To avoid difficulties for the global HMM analysis, traces were simulated with time-independent, normally-distributed noise. From the three-color data, we analyzed the yellow channel alone (1-color), yellow and red together (2-color) as well as the full three-color data (3-color). As the ground truth is known, we were able to initialize a three-color HMM analysis such that it could eventually converge. Whereas local HMM struggles to analyze the traces yielding an almost random guestimate of the state, both global HMM and Deep-LASI perform similarly well in all cases. As more channels become available, the state classification becomes more accurate.

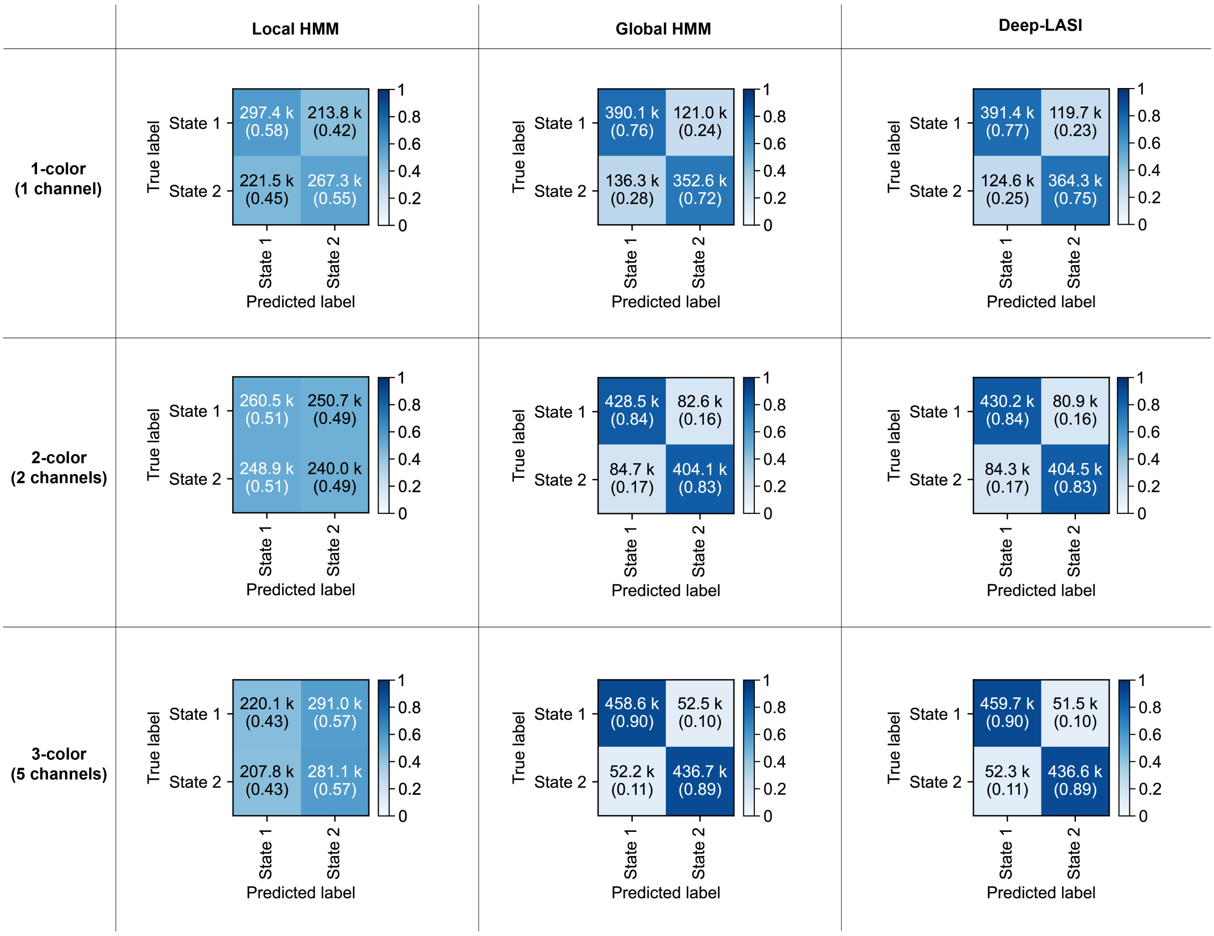

Supplementary Figure 3.6: Confusion matrices of frame-wise state predictions for local HMM, global HMM and Deep-LASI, performed on a global data set containing 2000 traces with 500 frames each. Three-color traces were simulated and the yellow channel with yellow excitation (1-color), yellow and red channels with yellow excitation (2-color) and all five channels for the three-color data with blue and yellow excitation were analyzed (3-color). In all cases, local HMM is unable to learn states and transitions due to the limited amount of training data. Global HMM and Deep-LASI are both able to effectively remove background noise, showing similar accuracies. All models show increased performance with higher number of channels due to effectively decreasing the signal-to-noise ratio. The data set was simulated based on 3-color FRET using the following parameters: Two states with only the yellow dye transitioning between E(YR) = 60 % and E(BY) = 40 % to E(YR) = 40 % and E(BY) = 60 %, a static blue to red FRET efficiency with E(BR) = 20 %, a symmetric transition probability of 0.05 per frame, and

the addition of normally distributed noise resulting in a mean FRET distribution width of 0.23 (averaged over the yellow-red FRET efficiency states).

3.6. Training and validation loss

The training and validation loss for all pre-trained deep-neural networks are shown in Supplementary Figure 3.7. Of the ~200,000 traces generated for training, ~160,000 were used in each epoch for training and then the capacity of the network to generalize what it learned was tested with the remaining ~40,000 traces. The error was calculated using the categorical cross-entropy, i.e. the loss function:

$$Loss = -\sum_{i}^{C} t_i \log(f(s)_i) \qquad \textbf{Eq. 3.1}$$

where C is the total number of classes, t is the target vector and $f(s)$ is the one-hot encoded vector of scores.[11] The categorical cross-entropy is specifically designed for multi-class classification problems, where each input belongs to exactly one class out of multiple mutually exclusive classes. It calculates the dissimilarity between the predicted class probabilities and the true class labels, providing a measure of how well the model captures the correct class assignments. Therefore, the model is able to produce probabilistic outputs in the form of class probabilities. By optimizing the model to minimize the cross-entropy loss, it learns to assign higher probabilities to the correct class and lower probabilities to the incorrect classes. During training of the model, the loss should decrease but maintain similar values for the training dataset as for the validation set. If a lower loss is observed for the training dataset, then for the validation dataset, the network is overfitting (i.e. it is memorizing the traces rather than learning the features of the categories). All models show no or a minimal amount of overfitting. While the full number of epochs are displayed in each plot, the model with the lowest validation loss and lowest amount of overfitting was saved and implemented (indicated with an arrow).

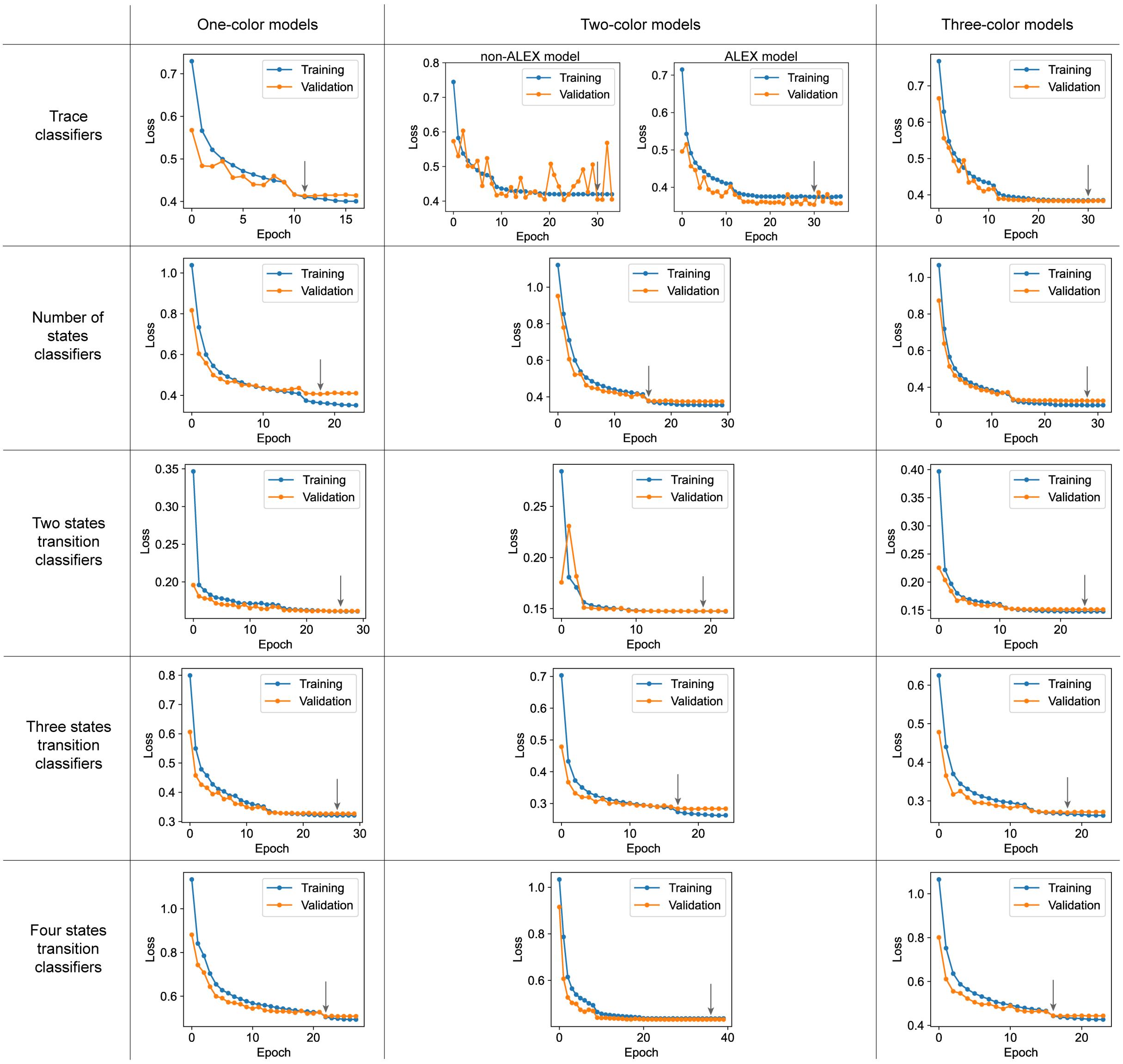

Supplementary Figure 3.7: Training and validation loss of all Deep-LASI models. Each row refers to the type of classifier and each column refers to the corresponding data type. For two-color data, there are two trace classifier models, one for ALEX and a second for non-ALEX measurements. Black arrows mark the saved model used when following epochs did not decrease the validation loss and indicated overfitting.

3.7. Analysis of kinetic data from the kinsoft challenge

We tested the performance of Deep-LASI on datasets provided by a recently published multi-laboratory software comparison study for extracting kinetics from smFRET data. As we contributed to this study using conventional HMM, we chose to analyze the datasets that did not require additional human input for interpretation of the data. The results are shown in Supplementary Figure 3.8. Deep-LASI returned values corresponding to the ground truth for the simulated dataset and close to the average values obtained for the experimental dataset.

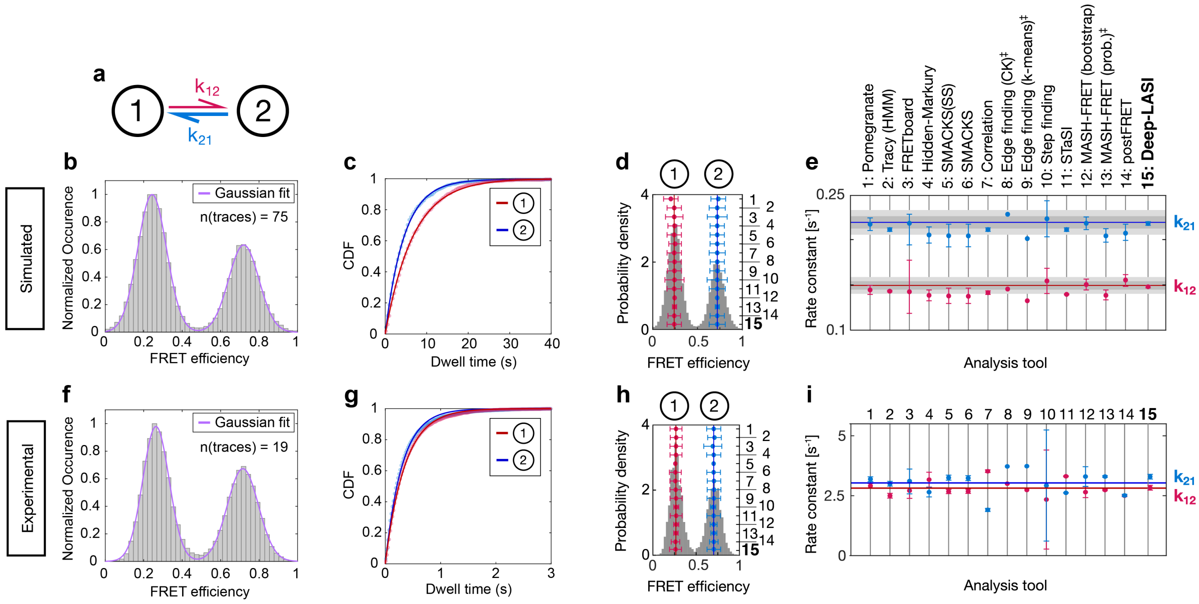

Supplementary Figure 3.8: Kinetic analysis of datasets from the kinetic software challenge. (a) An illustration of the kinetic two-state model connected by forward and backward rate constants: k_{12} and k_{21}. **(b)** A framewise FRET efficiency histogram (gray) of the simulated data extracted by the trace classifier. A Gaussian fit to the two populations are shown in magenta. **(c)** A mono-exponential dwell time distributions of the data in (b) obtained from the state-transition classifier. **(d)** The ground truth FRET histogram (gray) with state assignments labeled at the top and the inferred average FRET efficiencies in red and blue. Numbers on the right axis refer to the analysis tools specified in (e). Vertical lines indicate the mean over all tools. The error bars represent the standard deviations returned from the different analysis routines. **(e)** Rate constants and uncertainties inferred from the dataset in (d) by different labs using the respective analysis tools. The ground truth (GT) is indicated by the horizontal red and blue lines, the intrinsic uncertainty of the dataset is represented by dark gray (1σ) and light gray (2σ) intervals. **(f)** A framewise smFRET efficiency histogram (gray) of the experimental data extracted by the trace classifier. **(g)** The dwell-time distributions and corresponding mono-exponential fits of the data in (f) obtained from the state-transition classifier. A Gaussian fit to the two populations are shown in magenta. **(h)** A smFRET histogram of preselected traces from panel (h) where photobleaching and photoblinking contributions have been removed. State 1 is labeled in red and state 2 in blue. The vertical lines indicate the average value returned from analysis routines 1-14. The legend for the analysis routines is given in (e). The error bars represent the standard deviations returned from the different analysis routines. **(i)** Inferred rate constants from the experimental dataset in (h). The respective analysis tools are specified in (e). Horizontal red and blue lines indicate the mean of the inferred kinetic rate constants from analysis tools 1-14. The legend for the analysis routines is given in (e)

SUPPLEMENTARY NOTE 4: DEEP-LASI VERSUS MANUAL ANALYSES

4.1 Comparison of Deep-LASI and manual analysis of 2-color DNA origami traces |

We investigated the disagreements between Deep-LASI and manual classification and summarize the primary causes, illustrated through specific examples in Supplementary Figure 4.1. First, traces that exhibit non-ideal behavior after photobleaching are often thrown out by Deep-LASI as the entire traces is than categorized as an artifact whereas users may ignore characteristics of the traces in the non-accepted regions. Secondly, in cases where the leading frames are photobleached and the molecules reactivate, Deep-LASI tends to classify the entire trace as an artifact instead of extracting potentially useful information from the middle section of the trace. This is due to the fact that the training dataset does not yet include valid single molecule FRET traces starting with inactive dyes. Thirdly, Deep-LASI categorizes high noise or intensity fluctuations in the acceptor channel as noisy or classifies them as dynamic, but with insufficient confidence (> 70%) to include in further analyses. In such cases, the user has the flexibility to adjust the confidence threshold or consider the unfiltered dynamic category based on the maximum confidence output. Lastly, Deep-LASI may select traces that were discarded by manual evaluation, particularly short traces with fast dynamics that may be overlooked or considered noisy by the user.

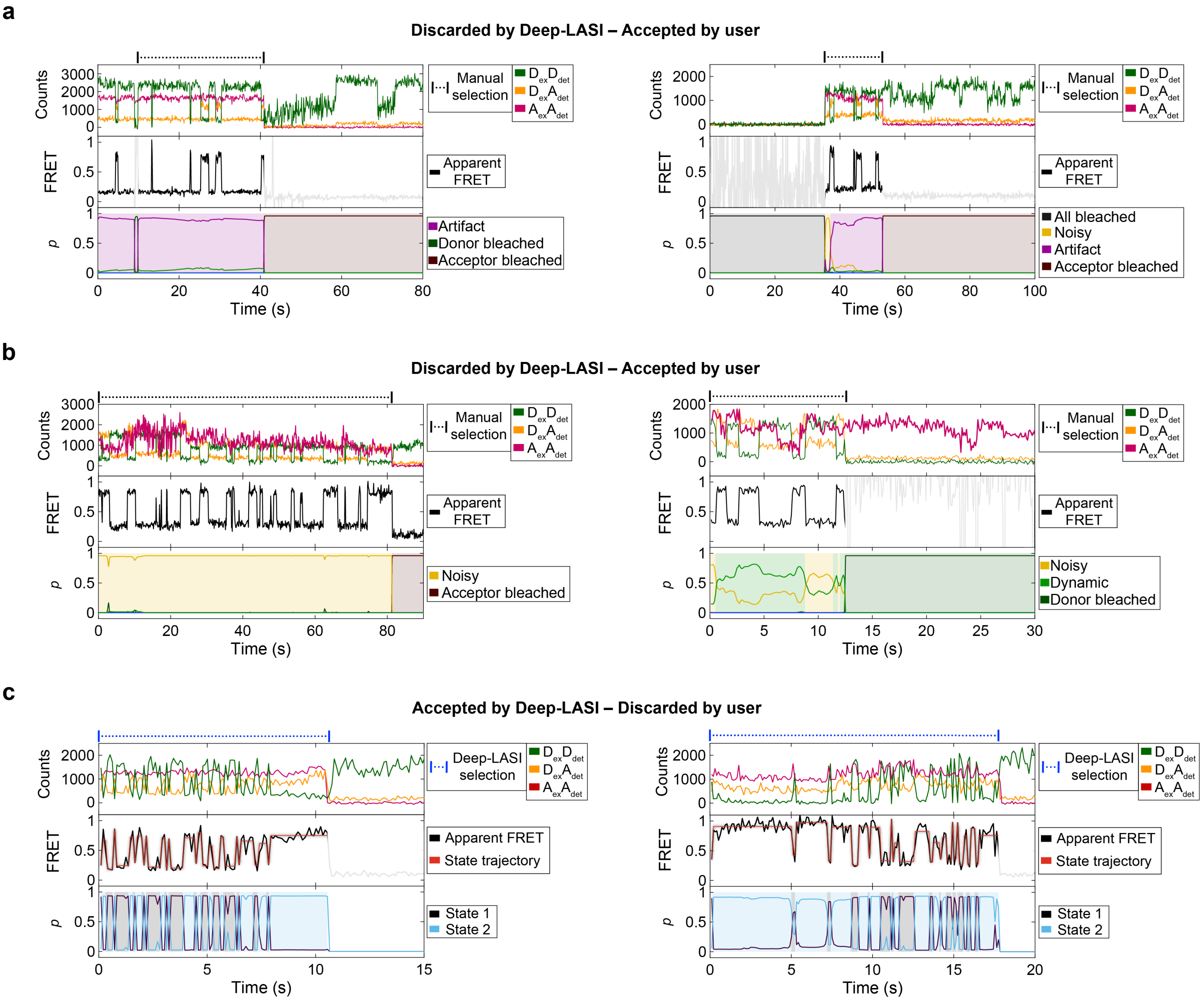

Supplementary Figure 4.1: Representative 2-color DNA origami traces with disagreements between manual and Deep-LASI selection. (a) Non-ideal donor signals. In the case of non-ideal donor intensities after photobleaching of the acceptor (left panel) or leading photobleached frames (right panel), Deep-LASI tends to classify the whole trace as an artifact while the manual selection still includes the middle region between the photobleached frames. (b) Non-ideal acceptor signals. Even though the FRET efficiency trace looks ideal, due to the high noise or intensity fluctuations in the acceptor channel, Deep-LASI classifies the trace as noisy (left panel) or is not confident enough (> 70 %) to classify the trace as dynamic (right panel). In the latter case, the user could influence the selection of this trace by lowering the confidence threshold. (c) Traces selected by Deep-LASI that were discarded by manual evaluation. Short traces (pay attention to the timescale on the x-axes) with fast dynamics can be either overlooked or deemed noisy by the user.

We further compare representative FRET traces analyzed using global HMM and Deep-LASI (Supplementary Figure 4.2). Global HMM tends to struggle in accurately capturing fast transitions, which can be attributed to the difficulties of HMM in distinguishing between fast transitions and noise or due to the inherent (non-Markovian) heterogeneities commonly encountered in single molecule experiments. Local HMM analyses can more easily deal with these heterogeneities. In comparison, Deep-LASI exhibits enhanced performance in detecting and characterizing these fast kinetics, suggesting its potential advantages over global analysis approaches.

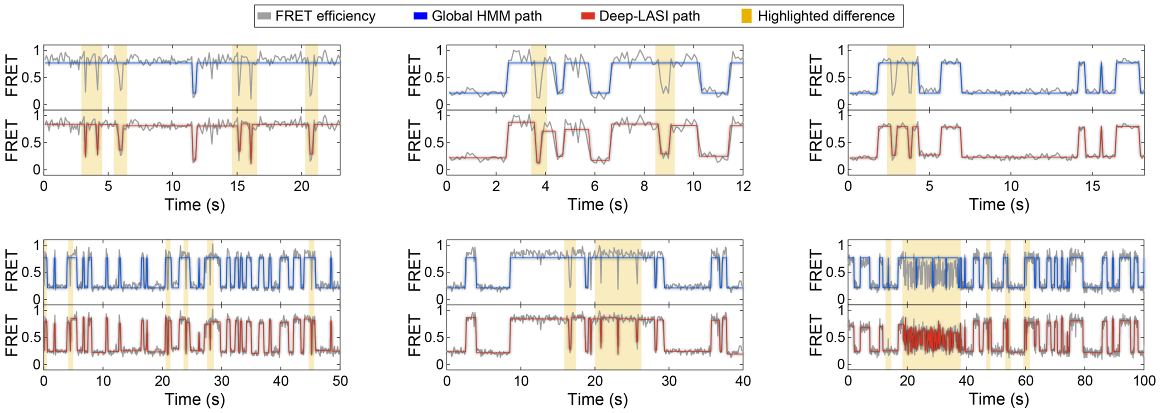

Supplementary Figure 4.2: Comparison of global HMM and Deep-LASI analyses. Representative 2-color DNA origami smFRET traces highlighting the differences between a global HMM and Deep-LASI analyses. In contrast to Deep-LASI, global HMM frequently misses fast transitions due to heterogeneities in the single molecule data.

4.2. Influence of different training datasets and comparison to user classification

The quality of a neural network rises and falls with the data by which it has been trained. To see the influence of different training datasets, we simulated three different datasets using the same parameters and used them to train Deep-LASI. The three different networks were then used to classify the two-color, two-state data shown in Figure 3. The results are shown in Supplementary Figure 4.3a. From the confusion matrix shown in Supplementary Figure 3.1, one would expect a consistency on the order of 95 %, provided the experimental data are similar to the training datasets. Each pair of networks agree within ca 93 %. Interestingly, the consistency between the neural networks is higher than that from two individual users (Supplementary Figure 4.3b). Here, user 1 tried to maximize the statistics and selected subsections of traces whereas user 2 was very conservative, only classifying the best traces as

dynamic. This suggests that neural networks may be more consistent in the analysis than different users.

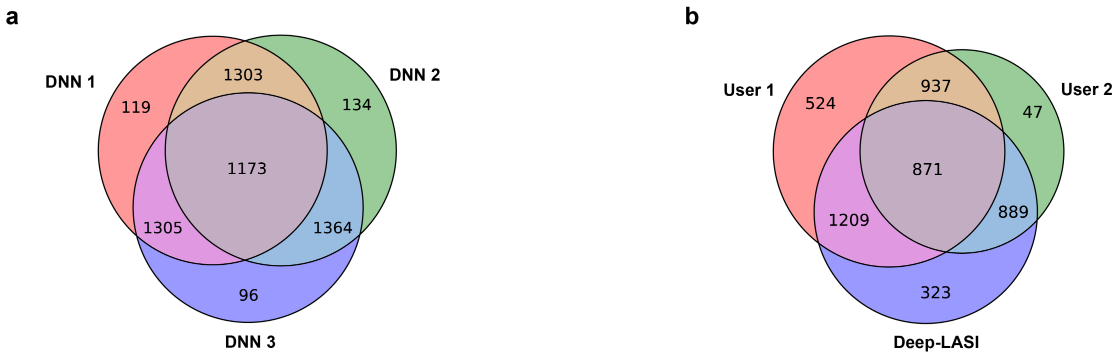

Supplementary Figure 4.3: Venn diagrams for differently trained neural networks and users. a) A Venn diagram showing the consistency between traces classified as dynamic (with a 70% confidence threshold) for three networks trained using different datasets (DNN1, DNN1 and DNN3) and applied to the experimental data shown in Figure 3. b) A Venn diagram comparing the number of traces classified as dynamic by two different users as well as DNN1 (with a 70% confidence threshold) for the same dataset used in panel a.

4.3 Deep-LASI versus manual analyses for 3-color DNA origami samples

We emphasize the importance of also using experimental data for testing deep learning methods trained on synthetic data since the simulations used for validation are usually generated by the same algorithm as the training dataset. Deep neural networks can easily learn biases of any kind in the training data, which may have no relationship to the respective category under new conditions. Hence, the prediction of categories with respect to ground truth simulations can produce high accuracies, which may not be directly translatable to real-world examples. Therefore, we compared the performance of our network models on real data with that of experts who manually analyzed the same dataset.

We benchmarked the three-color performance of Deep-LASI by comparing the automated analysis with traces manually selected by an expert user (Supplementary Note 5). We used the three-color L-shaped DNA origami structure with two binding locations spaced at 6 and 12 o'clock with complementary binding regions of 7.5 nt (Figure 4). Deep-LASI yielded 581 usable smFRET traces versus 694 for manual selection out of a total of 2545 extracted traces (Supplementary Figure 4.4a). The two uncorrected, framewise smFRET histograms are almost identical. The automatically extracted FRET correction factors, which are based on the predictions of the three-color trace classifier, were compared to those determined manually. The expert user selected the relevant regions of the traces for determining various FRET correction factors by hand. Very similar distributions and median values were obtained for the YR correction factors (Supplementary Figure 4.4b). For BR, both direction excitation and spectral crosstalk terms are small and the differences are not significant here. Due to the high stability of the yellow fluorophore, it is challenging to collect enough statistics to directly derive the detection correction factor. Hence, it is calculated from the product of the BY and YR γ factors. For BY, the distributions for spectral crosstalk from Deep-LASI and manual selection are consistent. However, for both direct excitation and the detection correction factor, there are

differences of ~15 %. Manual selection with the blue fluorophore is difficult because of the low fluorescence intensity of the blue dye. In the manually selected regions for direct excitation, a second population is visible due to difficulties of distinguishing between a Y only fluorophore and a dim B fluorophore undergoing high FRET. Similarly, there are differences in the detection correction factor distribution. As Deep-LASI has more flexibility in choosing relevant regions of the traces for determining the correction factors, it is most likely that Deep-LASI is more accurate in these cases. FRET correction-factor determination is a potential source of human bias in the analysis of smFRET data and we demonstrate here an advantage of using a well-trained neural network for automated analysis.

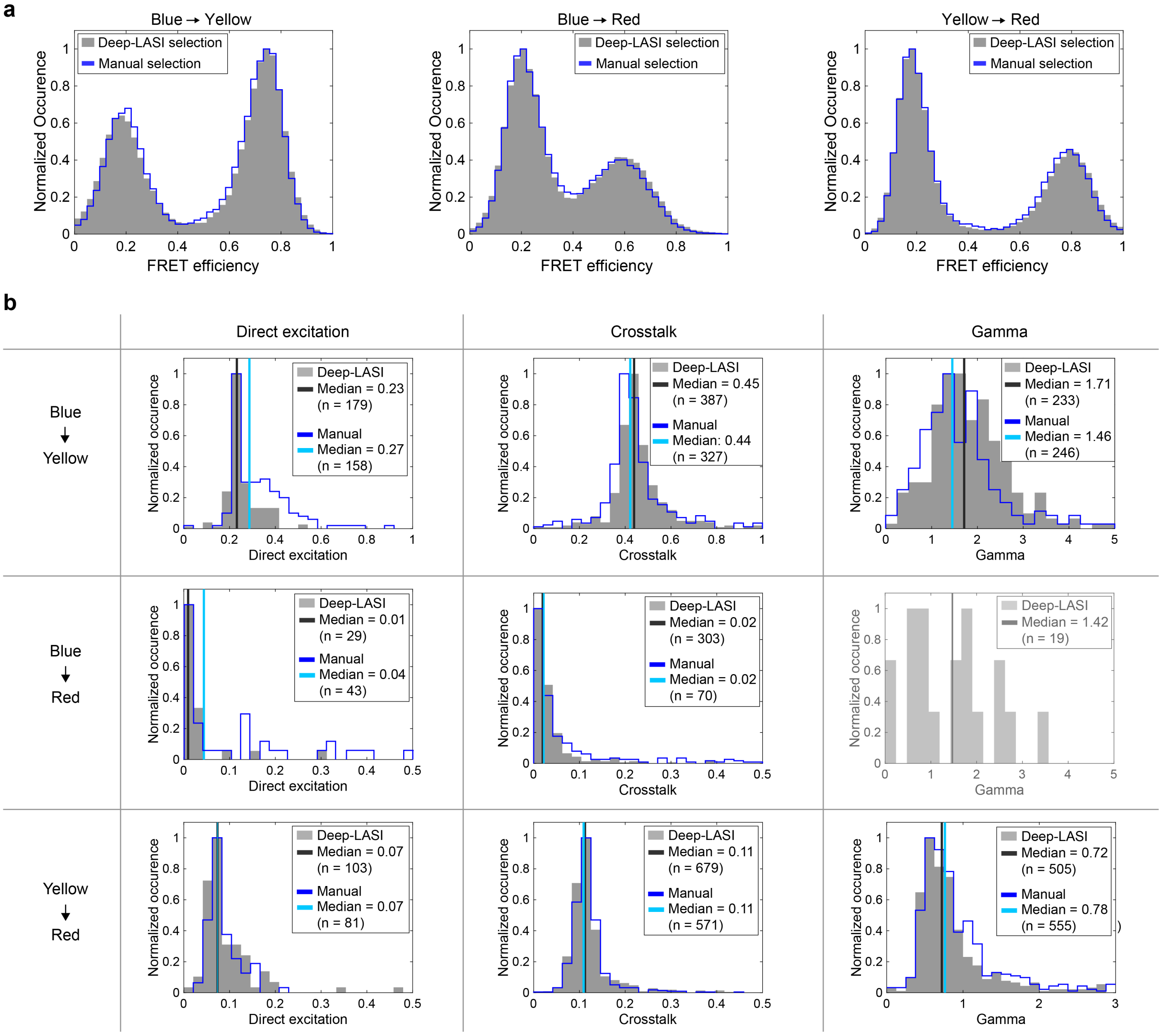

Supplementary Figure 4.4: Uncorrected smFRET histograms and correction factors extracted by Deep-LASI for the 3-color 2-state DNA origami. (a) Uncorrected framewise smFRET histograms for BY, BR and YR calculated from traces selected manually (n=694, blue line) versus the histograms determined by Deep-LASI (n=581, gray histograms). There is excellent correspondence between the histograms. **(b)** Each panel displays the normalized distribution of available correction factors from all traces categorized as ‘dynamic’ by Deep-LASI (gray filled histograms) or manually labeled as dynamic (blue histogram line). Due to the high stability of the yellow dye compared to the blue and red dyes, the number of usable traces to calculate blue/red detection correction factor was too low to be determinable. Therefore, we used the theoretical value of 1.23 (for Deep-LASI compared to 1.15 for manual selection) for the blue/red gamma factor determined from the product of the gamma factors for blue/yellow and yellow/red.

4.4 Comparison of 3-color DNA origami traces selected manually and/or via DNN classification.

To gain insights into the selection criteria of traces performed manually and via Deep-LASI, we examined the traces in detail that were selected differently. Examples are shown in Supplementary Figure 4.5. In general, similar differences arise as observed in the two-color FRET classifications (Supplementary Figure 4.1). In case of three-color FRET, blinking and dark states of the blue dye are inherently difficult to spot during manual inspection of the trace, whereas Deep-LASI predicts dark frames with high accuracy. Single-molecule traces often exhibit ambiguity. Deep-LASI tends to disregard traces displaying non-ideal intensities due to the way it was trained. Conversely, when manually selecting traces, users may incline towards including more non-ideal traces to improve the statistics and their selection can be subjectively influenced by a myriad of parameters.

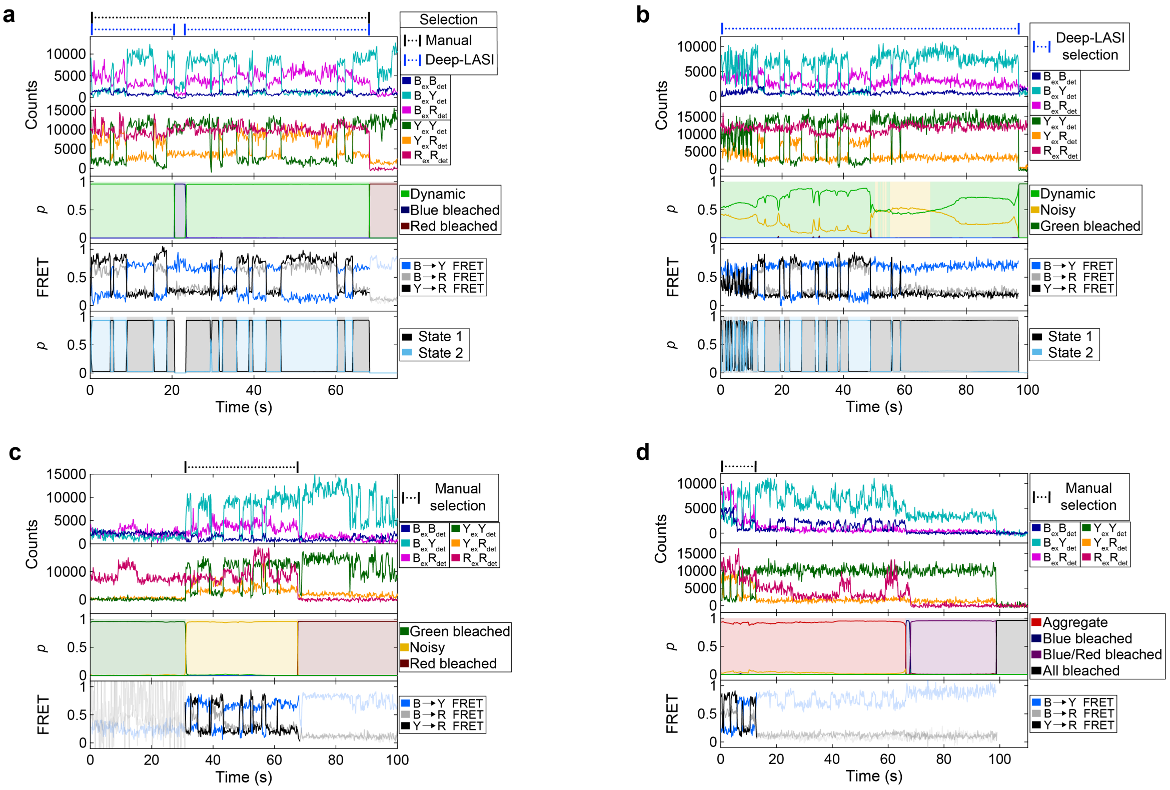

Supplementary Figure 4.5: Representative 3-color DNA origami traces with disagreements between manual and Deep-LASI selection. (a) Short blinking events. In this trace, Deep-LASI and manual selection agree in general but Deep-LASI excludes frames with an inactive blue dye. Blinking events of the strongly quenched blue dye in 3-color experiments can be easily missed during manual selection. **(b)** Regions of traces with high-noise or fast dynamics. The trace shown here was excluded manually due to seemingly high noise at the beginning of the trace. Deep-LASI predictions show the competing categories of 'dynamic' and 'noisy'. The summed confidence for the 'dynamic' classification exceeds the user defined threshold of 70 % and the state classifier predicts state transitions with high confidence. **(c)** Initial dark frames and non-ideal intensities. In the training datasets, we currently do not start with photobleached molecules the begin to fluoresce during the traces. Here is an example of Deep-LASI's tendency to classify valid sections as noisy due to leading bleached frames or erratic intensities during bleached frames. **(d)** Short traces. Short section in the beginning of the trace is manually selected whereas Deep-LASI classifies the whole trace as an aggregate due to intensity spikes in the acceptor channel after the valid section.

Supplementary Note 5: Manual Analysis of Single-molecule TIRF Data

5.1. Work-flow

We benchmarked the performance of Deep-LASI by comparing it to manually analyzed single-molecule data from an expert user. Starting from individual movies, the procedure for extracting the intensity information over time is highlighted in Supplementary Figure 5.1.

The procedure begins with:

(1) a pixel-wise mapping of the position between two or three cameras for two- and three-color experiments,

(2) camera-wise localization and excitation-cycle dependent assignment of intensities, and

(3) extraction of intensities and background correction for each detection channel.

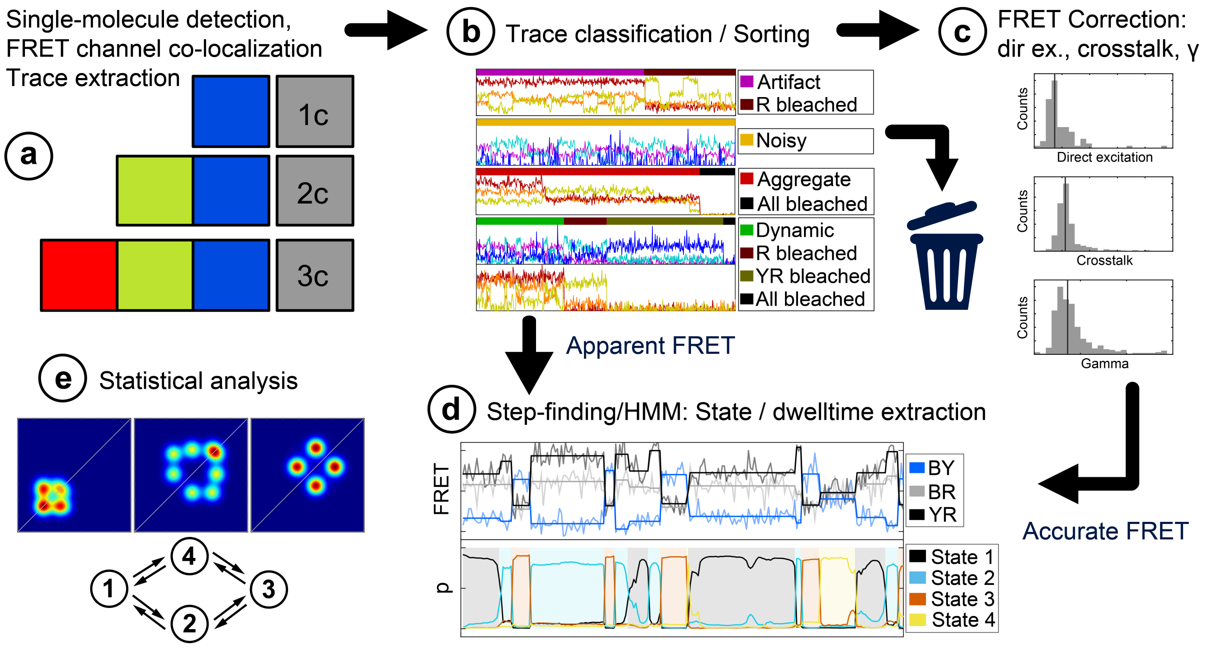

Supplementary Figure 5.1: Work-flow of data extraction, sorting, analysis and evaluation. a) One-, two-, or three-color data is collected with various excitation schemes and the time-dependent intensity traces extracted and corrected for background. b) The traces are then visually inspected and sorted either for further analysis or marked as junk. Regions of the trace can be selected for smFRET evaluation or for correction factor determination. c) After manual selection, trace-wise correction factors are extracted. d) For the dynamic traces selected for further analysis, the dwell-time distributions are determined using a Hidden Markov Model approach. e) From the HMM analysis, transition density plots are extracted from the smFRET data.

Next, the recorded traces were analyzed (Supplementary Figure 5.1b-e) either manually (Section 5.4-5.9) or assisted by neural networks (cf. Supplementary Note 1). Manual evaluation of single-molecule or multi-color FRET traces involves:

(4) the pre-sorting of traces suitable for 1c, 2c-, and 3c- smFRET analyses

(5) determining consecutive regions of the trace for evaluation and for determination of local and global correction factors

(6) Hidden Markov Modeling of smFRET traces to identify underlying states and dwell-times
(7) kinetic evaluation of transition rates and states using transition density plots (TDP), state-wise histograms and dwell-time analyses.

5.2. Camera mapping for FRET traces

In order to extract the fluorescent intensity traces of individual, fluorescently labeled DNA origami structures detected in various channels, an accurate localization and mapping of the detected emission channels across the three cameras or detection areas needs to be achieved. To compensate for potential chromatic aberrations and non-ideal alignment, an image transformation was used to map the corresponding pixels between different cameras/regions onto each other. The associated transformation matrix describing the potential shifts, tilts, etc. was obtained by imaging a calibration pattern on all three detection channels (Supplementary Figure 5.1a). As a calibration pattern, we typically use a zero-mode waveguide array.

5.3. Trace extraction and background subtraction

After mapping the different detection channels, the location of the individual single emitters needs to be determined and the intensity extracted. When msALEX excitation is used, the alternating laser excitation scheme needs to be taken into account and the intensity traces separated based on both the detection and excitation channels. The most blue-shifted detection channel serves as the reference channel (Channel 1). This refers to the blue excitation, blue channel (BB) for BY-, BR- and BYR-labeled samples or the yellow detection channel with yellow excitation (YY) for YR-labeled samples (Supplementary Figure 5.1a).

Individual molecules in the reference channel are identified by searching for the brightest spot in the summed projection of the movie. After calculating the central position of the molecule using a wavelet approach[12], the corresponding position in all other channels is calculated using the transformation matrix. Molecules in the projection images exhibiting detectable intensity in all desired channels are then selected. The intensities and background are extracted using different masks. For the signal, the pixels within an approximate circle of roughly 3 pixels radius around the central coordinates of the molecule are summed together. With a pixel size of 124 nm, the fluorescence signal of a single molecule is accumulated within an area of 614 × 614 nm^2. For the background, a mask representing roughly a circle with radius of 7.5 pixels (850 nm) and width of 2 pixel centered on the molecule is used. The background is calculated as the median value of all pixels inside the ring-shaped mask and averaged over a five-frame sliding window depending on the excitation cycle and the detection channel. Afterwards, the determined background is scaled to the signal mask and subtracted from the framewise intensity per each channel for each molecule. When analyzing single molecule traces from hand, trajectories which contain molecules within the background mask are discarded. In DeepLASI, these traces are typically discarded in the 'artifact' category during the first characterization step.

5.4. Manual trace selection and analysis

The background-corrected fluorescence intensity traces of the individual molecules are then inspected and sorted (Supplementary Figure 5.1b). The properties of the extracted traces are generally very heterogeneous. This stems from different sources including photochemistry, dye blinking, aggregates and impurities within the sample of different brightness. In all cases, molecules were rejected automatically if they exhibited (1) a low SNR or (2) a brightness that is significantly higher than expected for a single fluorophore (aggregates or impurities). We further classified traces according to

(1) their static and dynamic behavior
(2) the existence of photobleaching steps in the different intensity channels
(3) the order of bleaching steps between the different intensity channels
(4) the degree of labeling efficiency

With the presorted trajectories at hand, we next prepared the data either for (1) correction factor determination to obtain accurate FRET efficiencies (Supplementary Note 5.5) or (2) directly to kinetic and state evaluation based on background-corrected trajectories (1-color data) or apparent FRET efficiencies (2/3-color data). In the first case, we first derived the correction factors per trace and marked regions for trace evaluation by HMM afterwards (Supplementary Note 5.6). In the second case, we manually marked the regions in traces to be analyzed and added them to the 'HMM' category.

5.5. Accurate FRET determination

In real smFRET experiments, the intensity of the acceptor signal needs to be additionally corrected for direct excitation of the acceptor fluorophore and spectral crosstalk from donor into the acceptor channel. In addition, the one needs to correct for the difference in the detection sensitivity between the donor and acceptor fluorophores. The correction factors are denoted as:

de_{XY} for direct excitation of the acceptor fluorophore Y during excitation with X,
ct_{XY} for spectral crosstalk from the fluorophore X in the detector channel Y,
and γ_{XY} compensates for differences in detection sensitivities between channels.

We denote the background-corrected intensities as I_{XY} and the corrected Intensity as $I_{XY,corr}$, where x stands for the excitation source and y for the emission channel, i.e. $I_{BR,corr}$ denotes the background corrected emission of the acceptor within the red channel (R) after donor excitation in the blue channel (B).

Trace-wise and global correction factors

[13]Depending on when individual fluorophores photobleach, some of the correction factors can be extracted from the trace itself. However, in the vast majority of the traces, one cannot extract all correction factors individually. When a trace-wise correction factor is unavailable or unreasonable, the *median* value of the corresponding distribution of trace-wise correction factors for the particular correction factor is used to calculate the accurate FRET values, i.e. a global correction factor. Using traces that were presorted and categorized as 'Blue dye

bleached' (or 'yellow / red dye bleached', respectively), we first determined the trace-wise correction factors for direction excitation de_{XY} and spectral crosstalk ct_{XY}. Having corrected the background-corrected intensities against both contributions, we next determined the trace-wise correction factor γ_{XY}.

To derive the contribution of spectral crosstalk from the donor channel X in the acceptor channel Y, we determine the trace-wise correction factor ct_{XY} using the intensity information after photobleaching of the acceptor:

$$ct_{XY} = \left.\frac{\langle I_{XY} \rangle}{\langle I_{XX} \rangle}\right|_{no\ acceptor} \quad \textbf{Eq. 5.1}$$

Here, $\langle I_{XX} \rangle$ refers the mean donor intensity and $\langle I_{XY} \rangle$ to the mean acceptor intensity after donor excitation in the region of the trace where there is no acceptor fluorescence.

Similarly, we determined the correction factors for direct excitation of the acceptor during donor excitation using traces in which the donor fluorophore bleached first:

$$de_{XY} = \left.\frac{\langle I_{XY} \rangle}{\langle I_{YY} \rangle}\right|_{no\ donor} \quad \textbf{Eq. 5.2}$$

where $\langle I_{XY} \rangle$ and $\langle I_{YY} \rangle$ describes the mean acceptor emission after donor excitation or acceptor excitation, respectively.

Lastly, we determined the detection correction factors γ_{XY} compensating for differences in detection sensitivities between different channels. For this, we used traces where the acceptor photobleaches before the donor. The acceptor intensity is first corrected for direct excitation de_{XY} and spectral crosstalk ct_{XY}. We then derive the detection correction factor γ_{XY} per trace from the ratio of changes in donor and acceptor emission before and after photobleaching of the acceptor. The correction factors are denoted as:

$$\gamma_{XY} = \left.\frac{\langle \Delta I_{XY,cor} \rangle}{\langle \Delta I_{XX,cor} \rangle}\right|_{A\ bleaches} \quad \textbf{Eq. 5.3}$$

where $\langle \Delta I_{XX,cor} \rangle$ and $\langle \Delta I_{XY,cor} \rangle$ refer to the intensity difference for the mean donor and acceptor emission after donor excitation before and after acceptor photobleaching.

Data Correction

Once all correction factors are determined, every trace is corrected using the local, trace-wise correction factors, when available and suitable. Otherwise, the global correction factor is used. In three-color experiments, the corrected FRET efficiency for E_{YR} is calculated first since it is required for subsequent corrections. Upon yellow excitation, the same approach is used as for two-color FRET experiments:

$$I_{YY,corr} = I_{YY} \quad \textbf{Eq. 5.4}$$

$$I_{YR,corr} = I_{YR} - ct_{YR} I_{YY} - de_{YR} I_{RR} \quad \textbf{Eq. 5.5}$$

The corrected FRET efficiency is then given by the ratio of both corrected intensities

$$E_{YR} = \frac{I_{YR,corr}}{\gamma_{YR} I_{YY,corr} + I_{YR,corr}} \quad \textbf{Eq. 5.6}$$

For the BY FRET pair, the fully corrected intensities after blue excitation read as:

$$I_{BB,corr} = I_{BB} \quad \textbf{Eq. 5.7}$$

$$I_{BY,corr} = I_{BY} - ct_{BY} I_{BB} - de_{BY} I_{YY} \quad \textbf{Eq. 5.8}$$

The accurate BY FRET efficiency follows equation 5.5 with an additional term which takes into account the reduction in brightness of the yellow dye due to the FRET process between the YR pair:

$$E_{BY} = \frac{I_{BY,corr}}{\gamma_{BY} I_{BB,corr}(1 - E_{YR}) + I_{BY,corr}} \quad \textbf{Eq. 5.9}$$

The intensity of the red fluorophore after blue excitation needs to be corrected against direct excitation, contributions of both the blue and yellow dye due to crosstalk into the red channel and due to cascading of FRET from the blue dye over the yellow dye into the red channel:

$$\begin{aligned} I_{BR,corr} = I_{BR} - de_{BR} I_{RR} - ct_{BR} I_{BB} - ct_{YR}\left(I_{BY} - ct_{BY} I_{BB}\right) \\ - de_{BY} E_{YR} (1 - E_{YR})^{-1} I_{YY} \end{aligned} \quad \textbf{Eq. 5.10}$$

The accurate FRET efficiency of the BR FRET pair is then given by:

$$E_{BR} = \frac{I_{BR,corr} - E_{YR}\left(\gamma_{YR} I_{BY,corr} + I_{BR,corr}\right)}{\gamma_{BR} I_{BB,corr} + I_{BR,corr} - E_{YR}\left(\gamma_{BR} I_{BB,corr} + \gamma_{YR} I_{BY,corr} + I_{BR,corr}\right)} \quad \textbf{Eq. 5.11}$$

5.6. Hidden-Markov modeling

The kinetics and underlying states within the selected trajectories, i.e. either smFRET or intensity traces, were evaluated using Hidden Markov Modeling. The input data of both assays vary between 0 and 1. We anticipate that every molecule undergoes transitions between a fixed numbers of conformations described by a discrete number of states q_i (i = 1, ... Q). The behavior of the system can be captured by the joint distribution of the observed data $\mathbf{x} = (x_1, x_2, \ldots, x_T)$ and the corresponding hidden state sequence $\mathbf{q} = (q_1, q_2, \ldots, q_T)$. The joint distribution can be factorized as follows:

$$p(\mathbf{x}, \mathbf{q}) = p(x_1|q_1) \cdot p(q_1) \cdot \prod_{t=2}^{T} p(x_t|q_t) \cdot p(q_t|q_{t-1}) \quad \textbf{Eq. 5.12}$$

Here, $p(x_t|q_t)$ represents the conditional probability of observing x_t given the system is in the hidden state q_t, $p(q_1)$ represents the probability of being in the initial state, q_1, and $p(q_t|q_{t-1})$ represents the conditional probability of transitioning from state q_{t-1} to state q_t.

For a system with Q states in total, the transition probability matrix $\overline{\mathbf{K}}$ comprises $Q \times (Q-1)$ independent transition probabilities k_{ij} describing the likelihood for going from state i to state j. Here, it is a prerequisite for the Markovian process, that the row-wise sum of transition probabilities is normalized to 1. For a Hidden-Markovian process, the state sequence is not directly observable but buried in random noise of the system. It can only be inferred from measured observables **x**, i.e. the single-molecule trajectory, with a length of T data points. Here, the emission probabilities $f_{q_i}(x_t|\theta_q)$ serve as parameter to represent the relative likelihood for observing a specific FRET value (or intensity value) for a given set of model parameters θ_q and the molecule being in state q_i. For intensity measurements and single molecule FRET traces, it is appropriate to model the emission probability of a state q_i as a Gaussian distribution:[14,15]

$$f_{q_i}\left(x_t|\theta_q = \{\mu_{q_i}, \sigma_{q_i}\}\right) = \frac{1}{\sqrt{2\pi}\sigma_{q_i}} \cdot e^{-\frac{(x_t-\mu_{q_i})^2}{2\sigma_{q_i}^2}} \quad \textbf{Eq. 5.13}$$

The parameters estimators are: the mean value μ_{q_i} and covariance σ_{q_i}

$$\langle\mu_{q_i}\rangle = \frac{\sum_{t=0}^{T} w_{q_i,t} x_t}{\sum_{t=0}^{T} w_{q_i,t}} \quad \textbf{Eq. 5.14}$$

$$\sigma_{q_i}^2 = \frac{\sum_{t=0}^{T} w_{q_i,t} x_t^2}{\sum_{t=0}^{T} w_{q_i,t}} - \langle\mu_{q_i}\rangle^2 \quad \textbf{Eq. 5.15}$$

For this, we introduce the relative occurrence probability $w_{q_i,t}$, i.e. the conditional probability $w_{q_i,t}$ of being in state q_i given the data x_t at a time t, which is linked to the fraction of time spent in state q, W_q

$$W_q = \frac{1}{T}\sum_{t=1}^{T} w_{q_i,t} \quad \textbf{Eq. 5.16}$$

and emission probability $f_{q_i}(\boldsymbol{x}|\theta_{q_i})$.

$$w_{q_i,t} = \frac{W_q f_{q_i}(x_t|\mu_{q_i}, \sigma_{q_i})}{\sum_{i=1}^{Q} W_{q_i} f_{q_i}(x_t|\mu_{q_i}, \sigma_{q_i})} \quad \textbf{Eq. 5.17}$$

As equations 5.16 and 5.17 are recursive, we have to optimize them iteratively. To do this, we indirectly maximize the likelihood function by optimizing an expectation-maximization (EM) criterion function. For the EM criterion, we use the log likelihood function as it allows for an efficient estimation of the model parameters, even in cases where the likelihood function is intractable or difficult to optimize directly. The log likelihood function for determining a sequence of states **q** given the observed FRET trajectory **x**, is computed as the product of the emission probabilities weighted by the relative occurrence probabilities summed over all trajectories and is given by[15]:

$$\log L = \sum_{i=1}^{Q}\sum_{t=1}^{T} w_{q_i,t} \log\left(f_{q_i}\left(x_t|\mu_{q_i}, \sigma_{q_i}\right)\right) \quad \textbf{Eq. 5.18}$$

To optimize the log likelihood function, we employ the Baum-Welch algorithm, also known as the forward-backward algorithm. During each iteration of the Baum-Welch algorithm, an expectation step (E-step) and a maximization step (M-step) are performed. In the E-step, the algorithm calculates the expected values of the hidden states given the observed data and the current parameter estimates. These expected values are then used in the M-step to update the model parameters, aiming to improve the fit between the model and the observed data. The training of the HMM continues until the relative improvement in the log likelihood between consecutive iterations falls below a predefined threshold. In all of our HMM training procedures, we set the convergence threshold to 10^{-9}. To derive the transition density matrix, the HMM needs to be trained on the dataset to be analyzed. This working step can be carried out in two different approaches: the analysis can be carried out either (1) trace-wise or (2) globally. In the first case, the transition probability and emission probability are optimized for each individual trace while, in the second case, one uses a shared single transition probability matrix and parameters for the emission probability for all trajectories together. When analyzing 1-color and apparent FRET traces, we use a local HMM as the exact values of the states can be shifted due to the above-mentioned background contributions.

5.7. Parameters for Hidden Markov Modeling

FRET efficiencies of dynamic DNA Origami structures were conventionally analyzed by HMM using the HMM Pomegranate toolbox written by Jacob Schreiber (2016). Molecules were manually classified as dynamic or static, and time windows were selected for the data analysis. For molecules showing dynamic transitions, we choose an HMM model with two or three states, depending on the designed Origami structures. Start parameters were chosen assuming a self-adapting width μ, and a standard deviation σ of 0.05 with random uniform distribution. Convergence between the experimental data and the fit was assumed, when the change in likelihood during consecutive iterations was less than 10^{-9}. For visualization via TDP, each transition as superimposed as a 2D Gaussian function with a fixed width.

5.8. Evaluation of involved FRET states and interconversion rates

The last step involves the visualization of determined rates, i.e. dwell times, and states determined from the FRET and / or normalized intensity traces. We employed so called transition density plots (TDPs), which depict each transition that was identified by the HMM algorithm or the Deep-LASI state classifier in the recorded time traces as a single event in a 2D diagram. The diagram, hence, depicts and links the FRET value before and after an identified transition visually. In the case of 1-color data, we normalized the traces between the minimal and maximal value of observed counts of all measured single traces. The TDPs were generated as described by McKinney et al., i.e. all transitions are depicted as summed up two-dimensional Gaussian functions with an amplitude equal to the total number of transitions and a fixed variance of 0.0005.[14]

Supplementary Note 6: Details of Deep-LASI Analyses

6.1 Results for the three-color, two-state DNA origami structure with different binding site lengths

The three-color DNA origami structures were measured with four different lengths of complementary DNA for the two binding sites. The two binding sites contained the identical DNA sequence and lengths. The dwell time distributions determined from the state classifiers of Deep-LASI for the different three-color DNA origami structures are shown in Supplementary Figure 6.1. The same analysis workflow was followed for each sample: a fully automated categorization and prediction of state occupancy in traces labeled as 'dynamic' were performed with Deep-LASI followed by a manual selection of the different states and fit to a mono-exponential function:

$$f(x) = 1 - e^{-b \cdot x} \qquad \textbf{Eq. 6.1}$$

These experiments confirm that Deep-LASI is capable of extracting mono-exponentially distributed dwell times over a large range of kinetic rates.

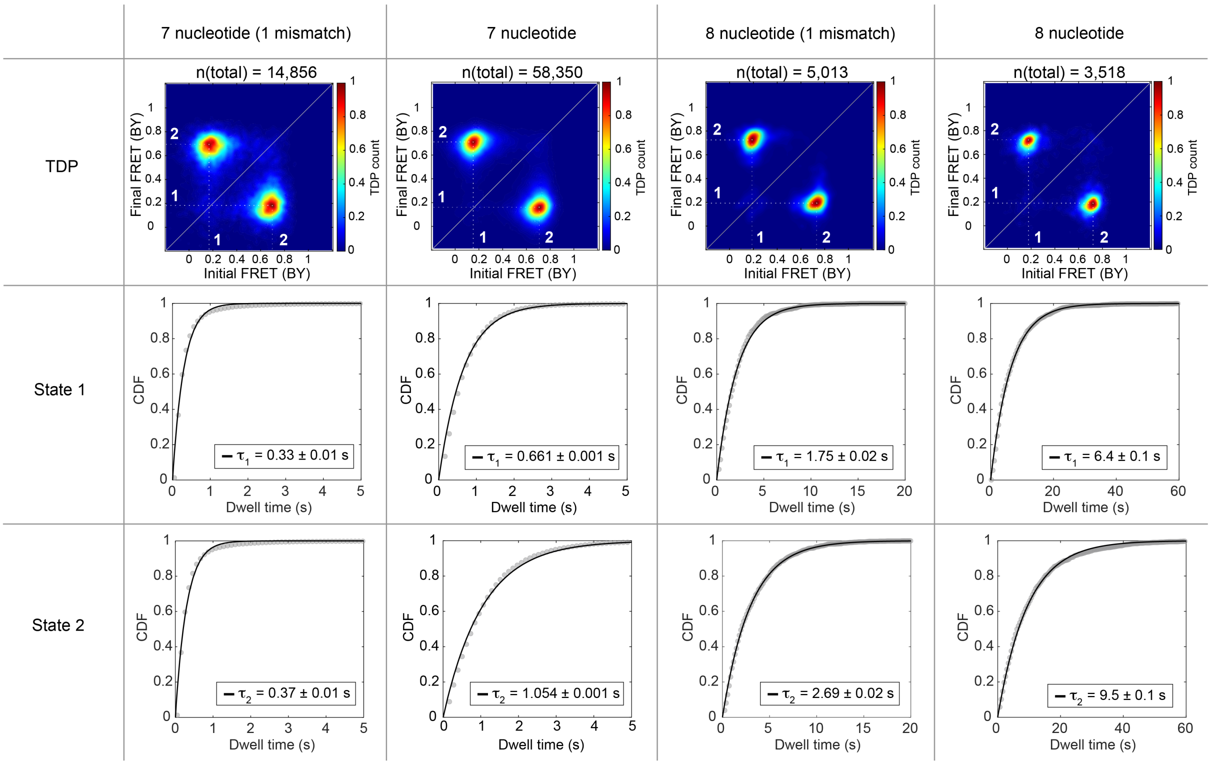

Supplementary Figure 6.1: Dwell-time distributions of the three-color, two-state DNA origamis with different binding site lengths. Each row corresponds to a specific state and each column depicts the TDPs (top) and dwell-time distributions (middle, bottom) extracted from the uncorrected blue-yellow transition density plots and fitted with a mono-exponential for each binding site length. The errors on the dwell times are the 95% confidence intervals returned by the fitting procedure (estimated from the Jacobian matrix).

6.2 Kinetics of the three-color, three-state DNA origami.

From the three-color, three-state DNA origami with 7 nt binding strands at positions 6 and 12 o'clock and a 7.5 nt complementary binding strand at 9 o'clock. Three populations were extracted automatically from the traces identified by Deep-LASI as dynamic. The dwell-time distributions of all 6 populations observed in the blue/yellow TDP plot (Figure 5c) were extracted manually and fit with an exponential function (Supplementary Figure 6.2). The dwell times of each state are in excellent agreement with the two-color, three-state DNA origami sample (Supplementary Figure 6.3), indicating that the additional blue dye in close proximity of state 2 does not influence the kinetic rates.

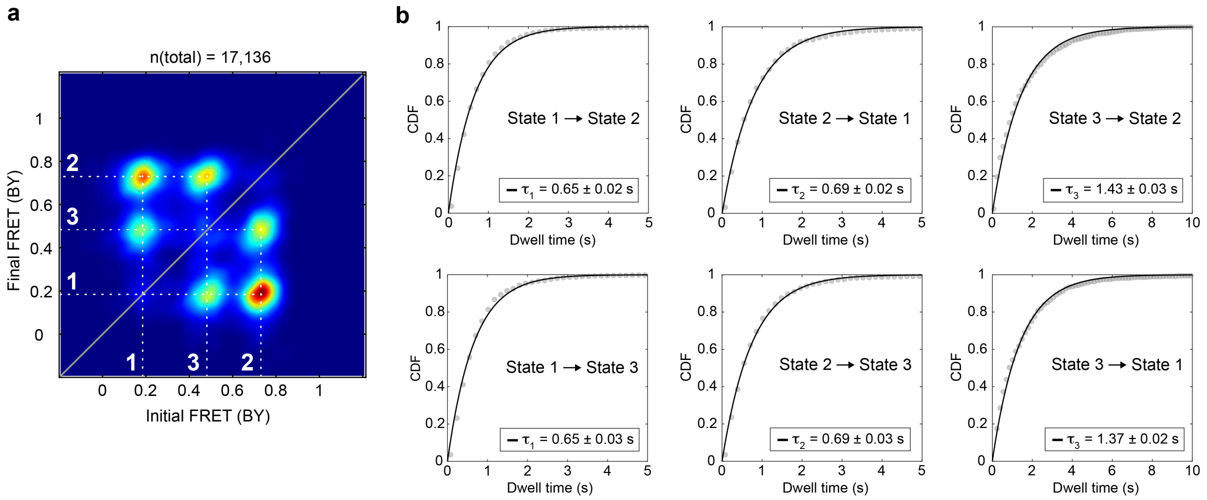

Supplementary Figure 6.2: Dwell-time distributions of the 3-color 3-state DNA origami. (a) The blue/yellow transition density plot and **(b)** the dwell-time distributions extracted from the BY-TDP and fit using a mono-exponential. The errors on the dwell times are the 95% confidence intervals returned by the fitting procedure (estimated from the Jacobian matrix).

6.3 Results for the two-color, three-state DNA origami structure

Next, we tested the performance of Deep-LASI on a more complex, two-color, multi-state system by introducing a third binding site on the DNA origami (Supplementary Figure 6.3a) and increasing the average transition rates. In contrast to the two-state system described above, State 1 and State 2 at the 6 o'clock and 12 o'clock positions are now characterized by 7 nt binding sites in the three-state DNA origami. The added State 3 at 9 o'clock has a 7.5 nt overhang. In the example trace shown in Supplementary Figure 6.3b, Deep-LASI extracts the dynamic section and identifies all transitions between the three states summarized in the TDP of apparent FRET efficiencies (Supplementary Figure 6.3c). As expected, the FRET efficiency of state 1 (0.83) and state 2 (0.21) do not change significantly compared to the two-state system. In addition, a third state with an apparent FRET efficiency of 0.31 is observed. However, as states 2 and 3 show a similar distance to the acceptor, the states and thereby the transitions are not easily separable. When looking at the dwell-time distributions, the transition out of state 1 is not affected by the degeneracy of states 2 and 3. However, the transition rates from state 2 or state 3 to state 1 differ significantly due to the different binding site lengths and can only be extracted using a bi-exponential fit (Supplementary Figure 6.3d):

$$f(x) = 1 - a \cdot e^{-b \cdot x} - c \cdot e^{-d \cdot x} \qquad \textbf{Eq. 6.2}$$

From the TDP, we can also extract the transitions between states 2 and 3. The transition from state 2 to state 3 can be well described by a mono-exponential distribution whereas the reverse transition from state 3 to state 2 has a second component due to the difficulties of clearly separating the different states.

From the single molecule trajectories, Deep-LASI also extracts the regions of the trace that can be used for determining the different correction factors. The FRET correction factor distributions determined by Deep-LASI are shown in Supplementary Figure 6.3e and are consistent with the correction factors of the two-state DNA origami dataset shown in Figure 3f. The framewise apparent smFRET histogram is shown in Supplementary Figure 6.3f (top, gray). In this histogram, states 2 and 3 merge into one degenerate state (0.27) due to heterogeneous broadening of the two populations. After correction (Supplementary Figure 6.3f, top, orange), the degeneracy is decreased and the low-FRET peak broadens. However, they are still not clearly separable. It is only after using the state-label information, which allows us to average the state FRET efficiencies that the two low-FRET populations become distinguishable and the individual FRET populations observed (Supplementary Figure 6.3f).

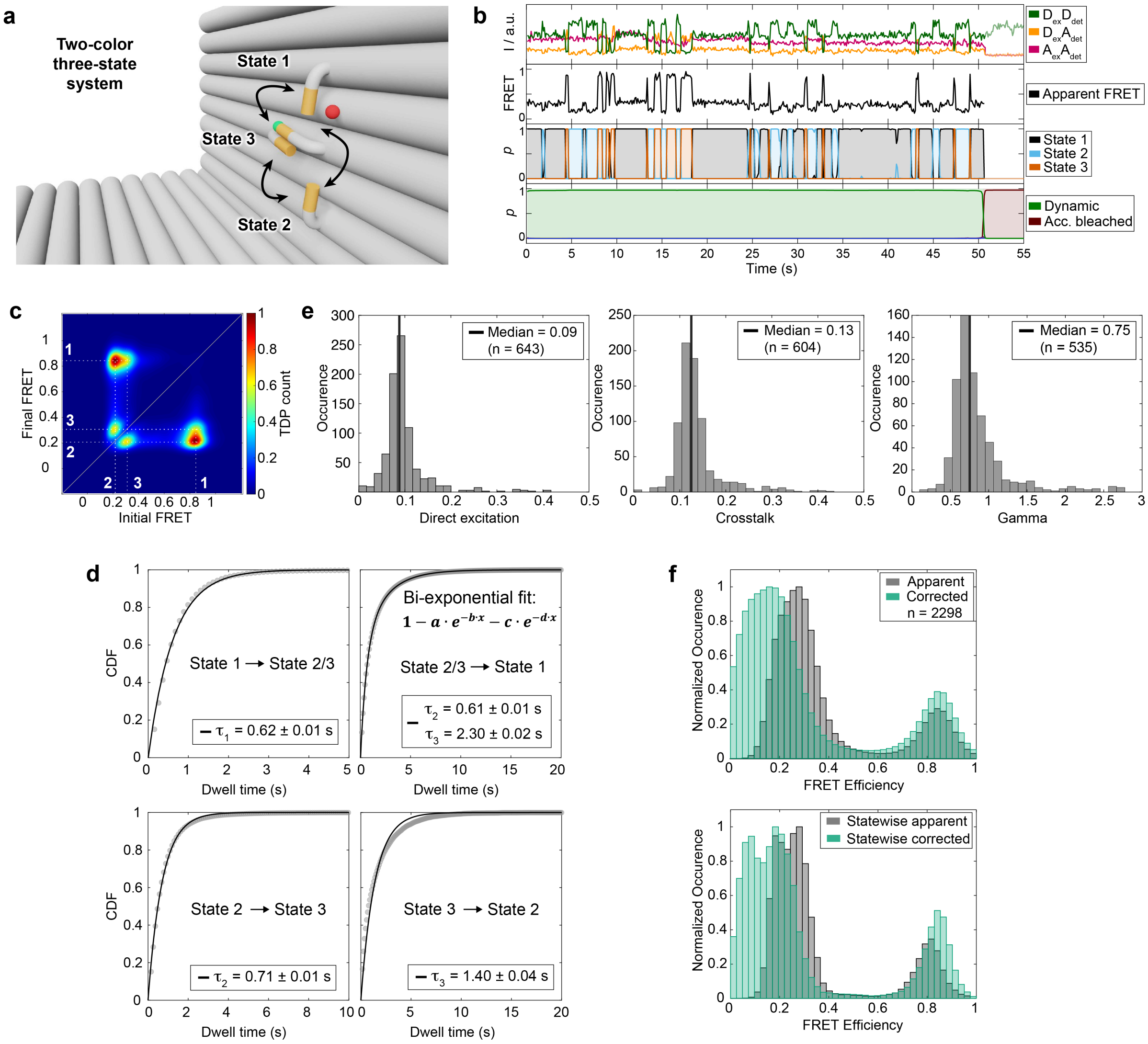

Supplementary Figure 6.3: Analysis of 2-color, 3-state DNA origami measurements. (a) Zoom-in of the L-shaped DNA origami structure with three binding sites. FRET is expected between a high FRET state 1 (12 o'clock), a low FRET state 2 (6 o'clock), and an intermediate FRET state 3 (9 o'clock). **(b)** A representative single molecule intensity trace and FRET trajectory. The upper panel shows the intensity in the yellow and red channels after yellow excitation and the red intensity after red excitation. The middle panel shows the corresponding FRET efficiencies for the dye pair. The third and fourth panels show the output of the Deep-LASI analysis for state-transition and trace classification respectively. **(c)** The TDP of the apparent FRET efficiency states are shown. Interconversion between three conformations with apparent FRET efficiencies of 0.21, 0.31 and 0.83 are observed. The three states are labeled in white. Total number of transitions: 174,697. **(d)** Exponential fits of the dwell time distributions for all states are plotted. The transitions from state 2 and 3 to state 1 were pooled together due to the high overlap and fit with a bi-exponential function. While the dwell time of state 2 in the bi-exponential fit is close to the dwell time extracted from the single population (state 2 to state 3), the dwell time of state 3 is significantly overestimated compared to the single population of transitions from state 3 to state 2. The errors on the dwell times are the 95% confidence intervals returned by the fitting procedure (estimated from the Jacobian matrix). **(e)** Correction factors for direct excitation, crosstalk and gamma extracted by Deep-LASI. **(f)** *top* Frame-wise weighted state-wise smFRET histograms of apparent and accurate smFRET efficiencies. A broadening of the low-FRET population is observed as the correction of the FRET efficiency begins to lift the degeneracy. *bottom* Plotting the framewise-weighted statewise smFRET histograms of apparent and accurate FRET efficiencies improves the contrast. Three peaks are now observable with corrected FRET efficiencies of 0.09 and 0.84 (in line with the two-state system), and a new third state at 0.19.

6.4 Kinetics as a function of Temperature

To investigate the influence of temperature on the binding kinetics, we used the two-color two-state L-shaped origami structure with minor changes (exchanged staple strands are noted with asterisks in Supplementary Table 7.2). Single molecule dynamics were measured between 19.3 °C and 25.0 °C. The transitions rates between state 1 and state 2 are given in Supplementary Figure 6.4. The rates change roughly by a factor of 2 per 2 °C.

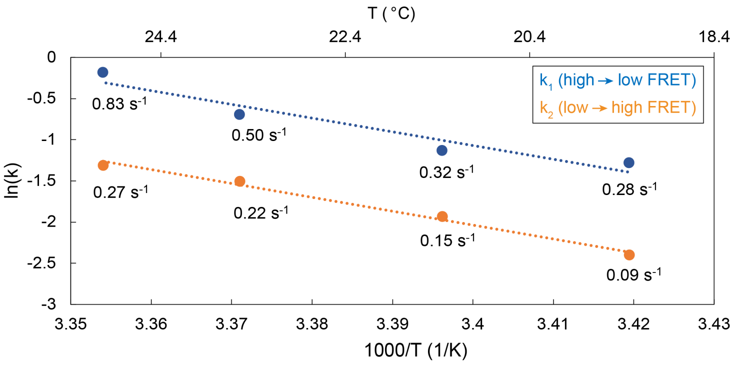

Supplementary Figure 6.4: Binding kinetics as a function of temperature. An Arrhenius plot of the transition rates between state 1 and state 2 of the two-color, two-state L-shaped DNA-origami structure. Linear fits (dotted lines) are shown to guide the eye.

6.5 Analysis of previous published 2-color Hsp70 Ssc1 using Deep-LASI

To test Deep-LASI on single-molecule FRET data from proteins, we reanalyzed data that we published previously[16]. These data were collected of proteins that were encapsulated in ~200 nm liposomes. Due to vesicle encapsulation, a photostabilization buffer could not be used. Hence, the protein data on this system had a lower signal-to-noise ratio than we typically had with the photostabilized DNA origami structures. In Supplementary Figure 6.5, we show examples of individual traces that were evaluated similarly or differently by the user and Deep-LASI. From these comparisons, we see that: 1) the user and Deep-LASI agree on traces that have sufficient SNR and otherwise show no anomalies, 2) manually selected dynamic traces with erratic intensities after photobleaching or broad FRET distributions tend to be categorized by Deep-LASI as noisy rather than dynamic, 3) Deep-LASI tends to include short traces that are trashed in the manual analysis and 4) traces that show clear features of overestimated background, multiple bleaching steps or other anomalies are discarded by both Deep-LASI and the user.

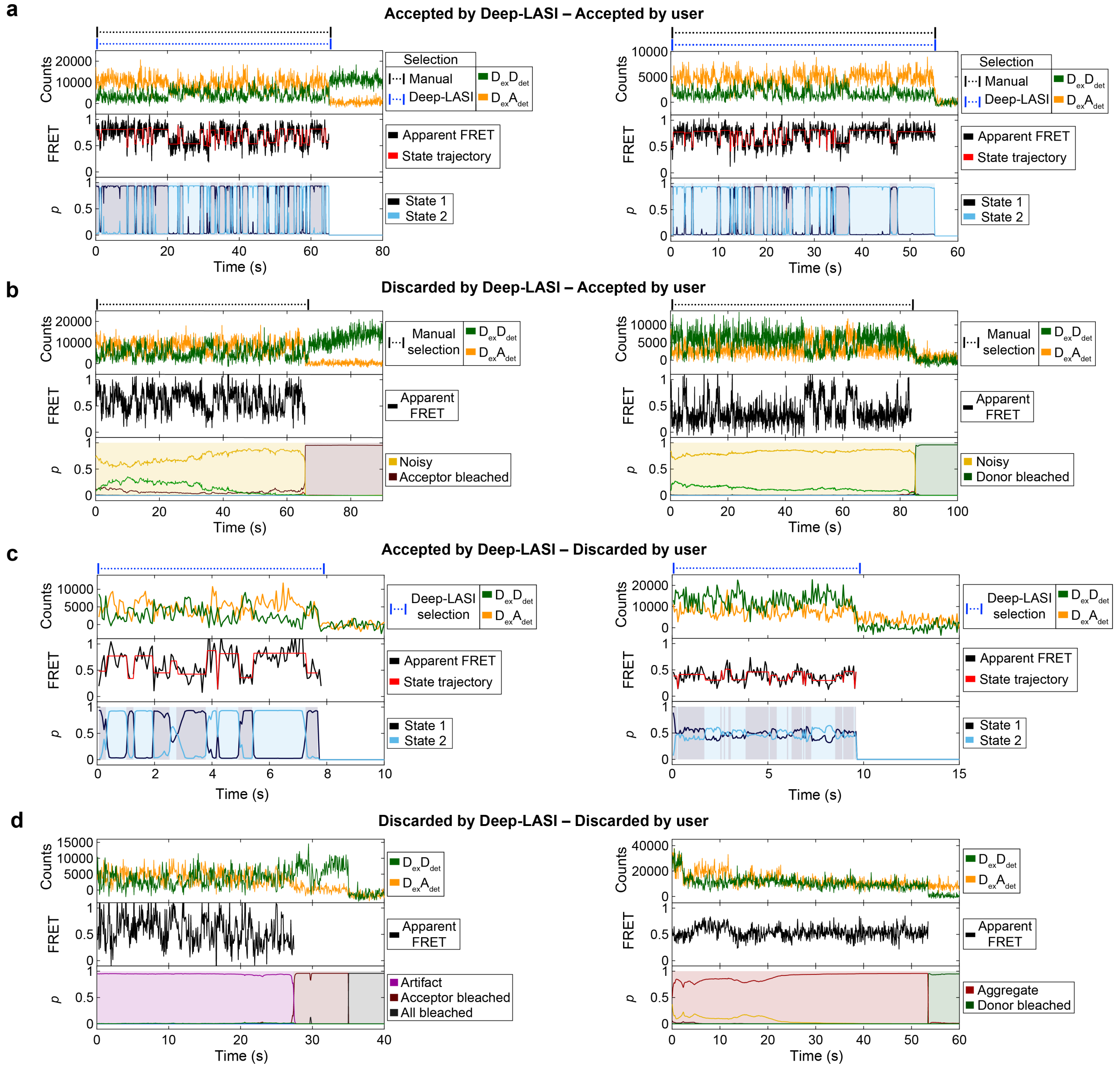

Supplementary Figure 6.5: Representative 2-color SSC1 traces with disagreements between manual and Deep-LASI selection. (a) Two true positive examples of manual and Deep-LASI selection agreeing on the validity of dynamic sections. The state trajectory (red) and confidence levels of the state transition classifier are shown in the two lower panels. **(b)** Two example traces that were included in the manual analysis but classified by Deep-LASI as noisy. The user could include these traces by lowering the confidence threshold of the 'dynamic' category. **(c)** Two examples of short traces classified as 'dynamic' by Deep-LASI but not selected manually. The left panel shows a valid dynamic trace, which was likely missed during evaluation. The right panel shows an apparent false positive classification by Deep-LASI. The low confidence of the state transition classifier (lower panel) allows this trace to be easily excluded the user. **(d)** True negative examples excluded from further analysis by both manual evaluation and Deep-LASI. The left panel shows a trace with overestimated background correction and artifact prediction. The right panel shows a trace two bleaching steps of the donor and aggregate prediction.

SUPPLEMENTARY NOTE 7: DNA SEQUENCES

Here, we describe the details of the L-shaped DNA origami structures. The structures were previously published by Tinnefeld et al.[17,18]. As a scaffold, we used the p8064 scaffold derived from M13mp18 bacteriophages. An overview of all designed DNA origami structures including name, the strand IDs of the introduced modified staple strands as well as the binding sites is given in Supplementary Table 7.1. The unlabeled staple strands are specified in Supplementary Table 7.2, staple strands with biotin modifications for surface immobilization are listed in Supplementary Table 7.3 and staple strands with fluorescent modifications for single-molecule FRET are summarized in Supplementary Table 7.3.

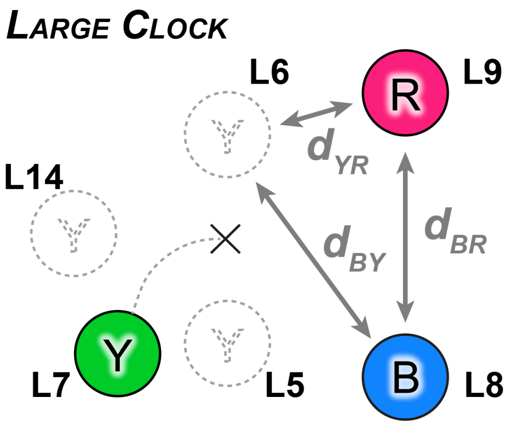

Supplementary Figure 7.1: Schematic of the replacement staple strands forming the 3 state, 3color FRET clock on the L-origami. The strands either carry one of the three fluorophores (L7, L8, and L9) or represent a binding site at 6, 9 and 12 o'clock (L5, L14 and L6 respectively).

The L-shaped DNA origami structures are made of 252 ssDNA staple strands annealed to a circular complementary ssDNA scaffold strand of 8064 nucleotides. The three fluorophores ATTO488, Cy3b and ATTO647N are introduced into the structures, by replacing the unlabeled ssDNA strands L7, L8 and L9 (Supplementary Figure 7.1) with strands containing the appropriate label (Supplementary Table 7.4). Binding sites for the L7-attached tether strands consisting of different lengths are introduced at position L5 and L6 for the 2 state systems with low and high FRET values and different binding rates, even with identical sequences. We refer to the binding site for staple strand L5 as 6 o'clock and staple strand L6 as 12 o'clock. For generating a 3 state FRET system, an additional binding site was introduced on staple strands L14 at 9 o'clock. In addition, for the implementation of the 9 o´clock binding site, the staple strands L12 and L13 are replaced by L12-13-I, L12-13-II and L12-13-III (Supplementary Table 7.4). All samples share biotinylated attachment sites at positions L1-L4 (Supplementary Table 7.3).

After folding and purification of the origami samples, the correct folding was confirmed via transmission electron microscopy (TEM) and atomic force microscopy (AFM) as shown in Supplementary Figure 7.2. They form compact structures of roughly 60×20×20 nm^3.

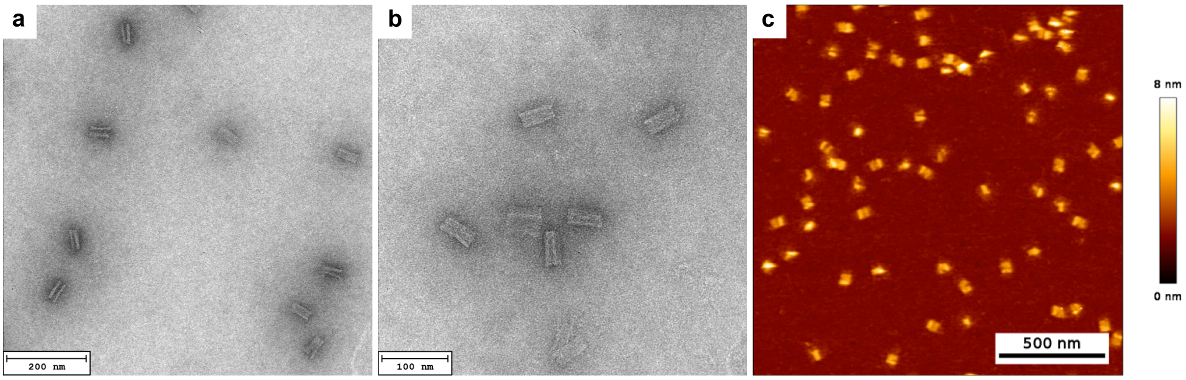

Supplementary Figure 7.2: Structural characterization of L-shaped DNA origami structures. (**a-b**) Transmission electron microscopy and (**c**) atomic force microscopy images indicated the efficient folding of the L-shaped DNA origami structures. Color table: Dark Gold.

AFM imaging was performed on a NanoWizard® 3 ultra AFM (JPK BioAFM AG/Bruker; Germany) in solution using 1x folding buffer. The DNA origami structures were immobilized on a freshly cleaved Mica surface (Quality V1, Plano GmbH; Germany) by Ni^{2+} ions, which were incubated on the Mica plate for 5 minutes with a 10 mM $NiCl_2$ solution. Afterwards, the mica was washed three times with ultra-pure water and dried by pressurized air. 10 µL of 1 nM DNA origami solution were then added and incubated for 5 minutes. Measurements were performed with a USC-F0.3-k0.3-10 cantilever (Nano World; Switzerland).

TEM imaging of the L-Origami structures was carried out on Ar-plasma cleaned TEM grids (Formvar/carbon, 400 mesh, Cu, TedPella, Inc.; USA). The DNA origami structures were stained with a 2 % uranyl formate solution. The imaging was performed on a JEM-1100 microscope (JEOL GmbH; Japan) with an acceleration voltage of 80 kV.

Supplementary Table 7.1. The applied nomenclature used for the designed L-shaped DNA origami structures with the corresponding staple strand IDs that carry the fluorescent dyes or the attachment of the pointer. The laser excitation scheme for the 3cFRET B-Y-R samples involves excitation at 488, 561 and 640 nm.

#	Name	Blue	Yellow	Red	Binding sites			Replaced
**1**	BYR-Pos6/12-6.5nt	488-L9	561-L7	640-L8	6.5nt-L5	6.5nt-L6	---	---
**2**	BYR-Pos6/12-7nt	488-L9	561-L7	640-L8	7nt-L5	7nt-L6	---	---
**3**	BYR-Pos6/12-7.5nt	488-L9	561-L7	640-L8	7.5nt-L5	7.5nt-L6	---	---
**4**	BYR-Pos6/12-8nt	488-L9	561-L7	640-L8	8nt-L5	8nt-L6	---	---
**5**	BY-Pos6/12-7.5nt	488-L9	561-L7	---	7.5nt-L5	7.5nt-L6	---	---
**6**	BR-Pos6/12-7.5nt	488-L9	---	640-L8	7.5nt-L5	7.5nt-L6	---	---
**7**	YR-Pos6/12-7.5nt	---	561-L7	640-L8	7.5nt-L5	7.5nt-L6	---	---
**8**	YR-Pos6/9/12	---	561-L7	640-L8	7nt-L5	7nt-L6	7.5nt-L14	L12-13-I-III
**9**	BYR-Pos6/9/12	488-L9	561-L7	640-L8	7nt-L5	7nt-L6	7.5nt-L14	L12-13-I-III

Supplementary Table 7.2. Unmodified staple strands used for the L-shaped DNA origami structure given from the 5' to 3' end. All oligonucleotides were purchased from Integrated DNA Technologies. *In the origami used for the measurements shown in Supplementary Figure 6.4, the staple strands L141, L153 and L165 were replaced with the sequences highlighted with the asterisks at the end of the table.

Staple ID	Sequence (5' to 3')
L1	ATCCAGAACAATATTAGTCCATCAGGAACGGT
L2	CGTGCCTGTTCTTCGCATCCAGCGCCGGGTTA
L3	ATAATCAGAAAAGCCCAACATCCACTGTAATA
L4	CATAGGTCTGAGAGACAAATCGTCGAATTACC
L5	AGAAACAGCTTTAGAAGGAAGAAAAATCTACGATTTTAAGCATATAAC
L6	GCACCCTCCGTCAGGTACGTTAGTAAATGAATAGTTAGCGTCAATCAT
L7	ACGATAAACCTAAAACAAAGAATACACTAAAACATTACCCAACAAAGC
L8	TGCTCATTCTTATGCGTTAATAAAACGAACTATATTCATTGGCTTTTG
L9	AAGGGAACCGGATATTCACTCATCTTTGACCCGTAATGCCATCGGAAC
L10	CGGAATCTCAGGTCTGTTTTAAATATGCATGCGAACGAATCATTG
L11	TGAATTACCAGTGAATGGAATTACGAGGCATATAGCGAGAGAATCCCC
L12	CATTATACGGTTTACCCATAACCCTCGAAATACAATGTTTAAACAGGG
L13	ATTCATATCAGTGATTTGGCATCAGGACGTTGTAACATAAACCAGACG
L14	TAATAAGAAGAGCCACCCTTATTAGCGTTTGCCATTCAACAATAGAAA
L15	GGCACCAAAACCAAAAGTAAGAGCAACACTATAGCAACGTAAATCGCC
L16	ATAAAAATATCGCGTTCTCCTTTTGATAAGAGCTATAT
L17	TACCAGTAACGCTAACAGTTGCTATTTTGCACCCCATCCT
L18	GAGGGTAGTTGCAGGGTGCTAAACAACTTTCACGCCTGGAAAGAG
L19	AGAGCCGCAAACAAATGAGACTCCTCAAGAGATTAGCGGGCAGTAGCA
L20	AGTTGATTAGCTGAAAAGAGTACCTTTAATTGTTAATTCGGACCATAA
L21	TCGATAGCAGCACCGTAAAATCACGTTTTGCT
L22	AAAGACAAATTAGCAAGTCACCAATGAAACCA
L23	ATATTCACCGCCAGCATTGACAGGCAAAATCA
L24	TTTTCCCTTACACTGGTTGC
L25	CTCCAATCGTCTGAAATTTT
L26	TTTTTGCCTGAGTAGAAGAA
L27	TTTTCCCGACTTACAAAATAAACAGTTTT
L28	ATACGCAAAGAAAATTATTCATTAAAGGTGAATTTT
L29	TTTTCTTTACAAACAATTCG
L30	TTTTAAGTTACCAGGGTAATTGAGCTTTT
L31	TTTTTAAACGATGCTGATGG
L32	TTTTCAGGGTGGTTTTTCTT
L33	ACAAAGTATGAGGAAGCTTTGAGGACTAAAGATTTT
L34	CCGAATCTAAAGCATCTTTT
L35	AGATGAAGGGTAAAGTTTTT
L36	TTTTCGCAAATGGTCAATAAACCATTAGATGC
L37	TCGAAGATGATGAAACTTTT
L38	AGAGCAAATCCTGTCCAGATACCGACAAAAGGTAATTTT
L39	TTCCGGAATCATAATTTTTT

L40	TTTTTGGATTATTTACAGAA
L41	TGCGGCCAGAATGCGGTTTT
L42	TCAGCAGCAACCGCAATTTT
L43	TTTTAGAGCGGGAGCTAGAT
L44	TTTTGCTAATATCAGAGAGATAACCCCGCCACCGCG
L45	ACCTCGTCATAAACATTTTT
L46	TTTTTTGAGGGGACGACGAC
L47	TTTTAACAGTACCTTTTACA
L48	TTTTTGGCCTTCCTGTATAA
L49	TTTTGGCGCATAGGCTGGCTAACGGTGTTAAATTGT
L50	TCACCGGAAGCATAAATTTT
L51	TTTTTATCATCGCCTGAACAGACCATTTT
L52	TTCATAGGGTTGAGTGTTTT
L53	TTTTTAGCCCGGAATAGCCTATTTCTTTT
L54	TTTTCCCTCAGAGCCACCACCCTCAGAAAGCGCTTA
L55	TAGTAATAACATCACTTTTT
L56	TTTTTTGTTCCAGTTTGGAACAAGA
L57	TTTTCGGGCCGTTTTCACGG
L58	TTTTATTGCTGAATATAATACATTTTTTT
L59	TTTTAGTAATTCAATCGCAAGACAATTTT
L60	TTTTGAATGCCAACGGCAGC
L61	CAGATGAATATACAGTTTTT
L62	TTTTCCATATTATTTATCCCAATCCAAAGTCAGAGA
L63	TTTTTTATCACCGTCACAGCGTCAGTTTT
L64	TTTTCTTTTTCACAACGGAGATTTGTTTT
L65	TTTTGTGTAGGTAAAGATTC
L66	TTTTTTTTTTTTAAAACTAG
L67	TTTTGATTAAGACGCTGAGA
L68	TTTGCGTATTGGGCGCTTTT
L69	ATTATAGCGTCGTAATAGTAAAATGTTTTTT
L70	TAGTCAGAAGCAAAGCGGATTTT
L71	TTTTTAGACTGGCATCAGTTGAGATTTTTT
L72	CATAATAATTCGCGTCTTTT
L73	ATATATATAAAGCGACGACATCGGCTGTCTTTCCTTATCATTTTT
L74	AAAACGGTAATCGTTTTTTT
L75	ACAAATTATCATCATATTTT
L76	TTTTTTCCTGATTATCACGT
L77	TTTTCATATAAAAGAAAGCCGAACATTTT
L78	TTTTGTGTAAAGCCTGGCGG
L79	TTTTAAACATCAAGAAAAAA
L80	AATGCAATAGATTAAGGGCTTAGAGCTTATTTT
L81	ACATAGCGATAGCTTATTTT
L82	TTTTTTGCATCAAAAGCCTGAGTAATTTT

L83	CTGATAGCCCTAAAACTTTT
L84	GAAAGGAGCGGGCGCTAGGTTTT
L85	TTTTGCCTCAGAGCATAAAGAAAATTAAGCAATAAATTTT
L86	TTTTACTGTAGCCTCAGAACCGCCATTTT
L87	TTTTACCGTTCCAGTAAGCGTCATACATGGCTTCAGTTAAT
L88	AGTGTGCTGCAAGGCGTTTT
L89	TTAATTAAACCATACATACATAAAGGTGGCAATTTT
L90	CCGTGCATCTGCCAGTTTTT
L91	TTTTTTAGGAATACCACAGTAGTAATTTT
L92	TTTTATTGGGCTTGAGATGGCCAGAACGATT
L93	TTTTGAACAACTAAAGGAACACTGATTTT
L94	TTTTACTAGAAAAAGCCTGTT
L95	TTTCGACTTGATCGAGAGGGTTGATATAAGTATTTT
L96	ATTTAGAAGTATTAGATTTT
L97	TTTTACCTTGCTGAACCAGG
L98	TTTTTCCAAGAACGGGTGCGAACCTTTTT
L99	TTTTACGCATAATGAGAATAGAAAGTTTT
L100	TTTTAGAACGCGAGAAAACTTT
L101	TTTTGTTTCGTCACCAGTACTGTACCGTAAT
L102	CATGTTTACCAGTCCCTTTT
L103	TTTTGGAATTTGTGAGAGAT
L104	TTTTATTAAGTTGGGTACGC
L105	TTTTGGAACCTAAGTCTCTGAATTTTTTTTTT
L106	TTTTATCGCCATTAAAAATA
L107	GAGCCGATATAACAACAACCATCGCCCTTTTTTT
L108	CGGCCTCGTTAGAATCTTTT
L109	TTTTGCGCTGGCAAGTGTAG
L110	TAGTTGCCAGTTGCGGGAGGTTTTGAAGATCAATAA
L111	ATGGCTACAATCAACTGAGAGCCAGCAGCAAATGAAAAACGAACCTAATGCGCTTGGCAGA
L112	TCATCAACAAGGCAAATATGTACCCCGGTTG
L113	TTCAAATTTTTAGAAAAAACAGGAGCAAACAAGAGAATCGATGAAGGGTGAGATATTTTA
L114	CAACTAATGCAGACAGAGGGGCAATACTG
L115	GTACTATGGTTGCTTTTTTAGACACGCAAATT
L116	TGTAGCTCAACATTTACCCTCGAAAGAC
L117	ATCAAAAAGTCATAAAACGGAACAACATTATCAACTTTAGTAGAT
L118	AACGTCAATAGACGGGGAATACCCAAAAGAACAAGACTCCGTTTTTAT
L119	GGAGGGAAGAGCCAGCAATCAGTAGCGACAGACCAGAACCGCCTC
L120	AGCGAACCAGAAGCCTGGAGAATCACAAAGGCTATCAGGT
L121	GCCCCCTGGTGTATCACCGTACTC
L122	TACAGGCATTAAATTAACCAATAGGAACGCCATCAAAGTCAATCAGAATTAGCCTAAATCG
L123	TATTTTTGAGAGATCTGCCATATTTCCTCTACTCAATTGA
L124	CATTGCCTGAGAGTCTTTATGACCATAAATCATTTCATTT
L125	CCAGCCAGCTTTCCGGGTAATGGGGTAACAAC

L126	ATCGGCAAAATCCCTTACGTGGACTCCAACGT
L127	CCTGCAGCCATAACGGGGTGTCCAGCATCAGC
L128	GGGCCTCTTCGCTATTACGTTGTACCTCACCG
L129	ACCCTCATGCCCTCATTTCTGTATGGGATTTAGTTAAAGCAGCTTGA
L130	GTTGTACCACCCTCATAAAGGCCGGAGACAG
L131	TCTTTAGGCTGAATAATGCTCATTAGTAACAT
L132	CTCTCACGGAAAAAGAACGGATAAAAACGACG
L133	ACGCCAGATGACGGGGCGCCGCTAGCCCCAGC
L134	TTAATTTCATGTTCTATAACTATATGTAAATGCTGATGTCAATAGAATCCTTGACAAAATT
L135	TTTCATCGAATAATATCCAGCTACAATACTCCAGCAATTTCTTTACAG
L136	AATAAGTTAGCAAAAACGCAATAATAACGAGAATTAAAAGCCCAA
L137	GACCGTGTGATAAATACAAATTCT
L138	ACAAGAACCGAACTGATGTTACTTAGCCGGAAAAGACAGCACTACGAA
L139	ATCAAACTTAAATTTCTGGAAGGGCCATATCA
L140	CGCTGGCACCACGGGAGACGCAGAAACAGCGG
L141	GAGAAACATTTAATTTTACAGGTAGAAAG
L142	CTGCGCGGCTAACTCACAATTCCACACAACATACGAGTACCGGGGCTCTGTGGGTGTTCAG
L143	CCGAGTAAGCCAACAGGGGTACCGCATTGCAA
L144	AAACGGCGCAAGCTTTGAAGGGCGATCGGTGC
L145	CAAAAGAATAAAATACCCAGCGATTATACCAAGCGCGAA
L146	CTTAATTGAGACCGGAAACAGGTCAGGATTAGAGGTGGCA
L147	GCCAGTGCGATTGACCCACCGCTTCTGGTGCC
L148	CCAGAATGGAGCCGCCAATCAAGTTTGCC
L149	CCCCCTGCGCCCGCTTTAGCTGTTTCCTGTGT
L150	GGAAACCAGGCAAAGCGTACATAAGTGAGTGA
L151	AAATCAACACGTGGCATCAGTATTCTCAATCC
L152	AGGAGGTGGCGGATAAGTATTAAGAGGCTAAATCCTCTACAGGAG
L153	GACAGATGGACCTTCATCAAGAGCCCTGAC
L154	CTGAGGCCAACGGCTACAGAGGTTTCCATT
L155	ATAACCTTATCAACAAAAATTGTATAACCTCC
L156	AAATCAGCTCATTTTTGTGAGCGAATAGGTCA
L157	CACAGACATTTCAGGGATCTCCAAAAAAAAGGTTCTTAAAGCCGCTTT
L158	TAATAGTATTCTCCGTGCATTAAATTTTTGTT
L159	CGTTGGTAGTCACGACGCCAGCTGGCGAAAGGGGGATATCGGCCTGCGCATCGGCCAGCTT
L160	CTTCTGACCTAAATTTGCAGAGGCCAGAACGCAATTTACG
L161	GCTGCGCAACTGTTGGCAGACCTATTAGAAGG
L162	AGAACGTTAACGGCGTAATGGGTAAAGGTTTCTTTGCGTCGGTGGTGCTGGTCTTGCCGTT
L163	TTAGTTTGCCTGTTTAGGTCATTTTTGCGGATAGGAAGCCGACTATTA
L164	AATTACATAGATTTTCAATAACGGATTCGCC
L165	AAACGGGGTTTTGCTACATAACGCCAAAAAAGGCTTGTAATCTTG
L166	TGCGAATAATAATCGACAATGTTCGGTCG
L167	TTATACTTAGCACTAAAAAGTTTGTGCCGCCA
L168	GCCGTCACAATATAAAGAAACCACCAGAAGGAGCGGACTCGTATTACATTTGTCAAATAT

L169	ATTGCGTTTAACAACATTTCAATTACCTGAGCAAAAGGGAGAAACAGGTTTAAGATGATGG
L170	GGAGCCTTCACCCTCAGAGCCACC
L171	CCAGCTTACGGCTGGAAACGTGCCCGTCTCGT
L172	TTCGTAATCATGGTCATCCATCAGTTATAAGT
L173	AACAGAGGTGAGGCGGCAGACAATTAAAAGGG
L174	TTGAGTAAGCCACCCTCAGAACCG
L175	CAGTATGTTTATTTGCGAAGCCCTTTTTAATTGAGTTCTGAACA
L176	GCCTGTTTGCTTCTGTTACCTTTTAACGTTAA
L177	ATAAACAATCCCTTAGTGAATTTATCAAAAT
L178	CAAAGGGCCTGTCGTGTGGCCCTGAGAGAGTT
L179	CTCAAATGTTCAGAAATGGAAGTTTCACGCGCATTACTTCAACTGGCT
L180	CCCGCCGCGCTTAATGAAAGCCGGCGAACGTG
L181	TTCACCAGGTAGCAATGGCCTTGCTGGTAAT
L182	GTCGAAATCCGCGACCTGCTCCACCAACTTTTAGCATTC
L183	TGATTGCTTTGAATACAAACAGAATGTTTGGA
L184	TTCTGAAACATGAAAGTGCCGGCCATTTG
L185	AACCGTTTCACACGGGAAATACCTACATTTTGACGCTAAACTATCACTTCTTTAACAGGAG
L186	CGTTGAAAATAGCAAGCCCAATA
L187	CTTTTGCGTTATTTCAATGATATTCAACCGTT
L188	AAATCCCGTAAAAAAACGTTTTTTGGACTTGT
L189	TATCATTTTGCGGAACATCCTGATATAAAGAA
L190	AAATTATTTGGAAACAGCCATTCGAAAATCGC
L191	GCAGCAAGCGGTCCACAAGTGTTTTGAGGCCA
L192	CCAACATGACGCTCAATGCCGGAGGAAATACC
L193	TATTTTGTTAAAATTCGGGTATATATCAAAAC
L194	TGTTGCCCTGCGGCTGATCAGATGCAGTGTCA
L195	TGCGGGATAGCAGCGACGAGGCGCAGAGAAACGGCCGCGGTAACGATC
L196	TACCGATAGTTGCGCTTTTTCA
L197	TCAAATCACCATCAATACGCAAGG
L198	GTAAGAATAGTTGAAACTTTCGCAAACACCGC
L199	ATTGCCCTTCACCGCCCCAGCTGCTTGCGTTG
L200	AAGCGCATAAATGAAACAGATATAGAAGGCTTAGCAAGCCTTATTACG
L201	GGAATTAGGTAAATTTTCGGTCATAGCCCCACCGGAACCACCACC
L202	GTTTTCCCGTAGATGGCAGGAAGATCGCACT
L203	GCGAGAAAAGGGATGACGAGCACGTATAACGTGCTTTTCACGCTGAAGAAAGC
L204	GGGGCGCGCCCAATTCACTAAAGTACGGTGTCACGAGAATAGCTTCAA
L205	GAAATTGTTATCCGCTCACATTAAATTAATGA
L206	TTTTTAATGCACGTACAAGTTACCCATTCAG
L207	CAATTCATATAGATAATAAATCCTTTGCCCG
L208	CCTCAGAGCACAAGAAGAAAAGTAAGCAG
L209	CGCTCACTATCAGACGGTCCGTGAGCCTCCTC
L210	GCAGAGGCGAATTATTTTTCATTTGCTATTAA
L211	TTAGAGCTATCCTGAGGCTGGTTTCAGGGCGC

L212	GCCAGTACGTTATAAGGCGTTAAATAAGAATAAACACAAAT
L213	AACGTTATTAATTTTACAACTAATCAGTTGGC
L214	GCCGGGCGCGGTTGCGCCGCTGACCCCTTGTG
L215	CTGCAACAGTGCCACGTATCTGGTAGATTAGA
L216	TAAAGTTTAGAACCGCTAATTGTATCGCGGGGTTTAAGTTTGGCCTTG
L217	GAAACAACGCGGTCGCCGCACAGGCGGCCTTTAGTGACTTTCTCCACGTACAGACGCCAGG
L218	GTCCACTAAACGCGCGGACGGGCAACAGCTG
L219	GGAACCCAAAACTACAAACAGTTTCAGCG
L220	ATCGGCCTTAAAGAATAAATCAAAAGAATAGCCCGAGACCAGTGAGGGAGAGGGGTGCCTA
L221	ACAGTTGAGGATCCCCAGATAGAACTGAAAGC
L222	CCGGAACCGCAAGAAAGCAATAGCTATCTTACTCACAATCCGATTGAG
L223	GCAGTTGGGCGGTTGTCCAGTTATGGAAGGAG
L224	GCCGATTAAGGAAGGGCGCGTAACCACCACA
L225	TGTACTGGTAATAAGTTCAGTGCC
L226	CAAATCGTCAGCGTGGTGCCATCCCACGCAA
L227	TCTTACCATAAAGCCATAATTTAGAATGGTTTAGGGTAGC
L228	AGGCGAAAATCCTGTTGTCTATCACCCCCGAT
L229	GCCTAATTATCATATGATAAGAGATTTAGTTAATTTCAT
L230	TTTTCATCGGCATATTGACGGCACCACGG
L231	CTAGCTGATAAATTAACAGTAGGG
L232	CCCTGAACAAATAAGAAACGCGAGGCGTT
L233	CACATCCTCAGCGGTGGTATGAGCCGGGTCAC
L234	CAGGAAAAACGCTCATACCAGTAAATTTTTGA
L235	CCACCCTCTGTTAGGAAGGATCGTCTTTCCAGCAGACGATTATCAGCT
L236	CAAACCCTTTAGTCTTACCAGCAGAAGATAA
L237	GGCTTAGGTTGGGTTAAGCTAATGATTTTCGA
L238	CCGTCGGAGTAGCATTCAAAAACAGGAAGATT
L239	ATGAGTGACCTGTGCAGTTTCTGCCAGCACG
L240	CCGGCAAATCGGCGAAGTGGTGAAGGGATAG
L241	ACAAGAAATAGGAATCCCAATAGCAAGCAAATATAGCAGCATCCTGAA
L242	CCATTACCAAGGGCGACATCTTTTCATAGGCAGAAAGAATAGGTTGAG
L243	TGGAGCCGGCCTCCGGGTACATCGACATAAAA
L244	CACTCATGAAACCACCTTAAATCAAGATTGAGCGTCTTTTTGTTT
L245	GTATAAGCAAATATTTTAGATAAGTAACAACG
L246	AGGAAACCGAGGACGTAGAAAAAGTACCG
L247	CGGGAAACGAAAAACCTGATGGTGGTTCCGAA
L248	AGCATGTACGAGAACAATCCGGTATTCTAAGAACGATTTTCCAGA
L249	ACATTCTGAAGAGTCTCCGCCAGCAGCTCGAA
L250	GGGGTCATTGCAGGCGGGAATTGACTAAAATA
L251	TGCTTTCGAGGTGAATCTCCAAAA
L252	CAGTACCATTAGTACCCAGTGCCCGTATAAATTGATGAATTAAAG

*L141	TGCCCTGACGAGAAACATTTAATTTTACAGGTAGAAAG
*L153	GACAGATGGACCTTCATCAAGAGTAATCTTG
*L165	AAACGGGGTTTTGCTACATAACGCCAAAAAAGGCT

Supplementary Table 7.3. Modified staple strands given from the 5' to 3' end for the L-shaped DNA origami structures used. The biotin was used for surface-immobilization via a biotin/avidin interaction. All oligonucleotides were purchased from Biomers.

ID	Sequence (5' to 3')	Function	Replace
Bio-L1	**Biotin**-ATCCAGAACAATATTAGTCCATCAGGAACGGT	Attachment Biotin at 5'	L1
Bio-L2	**Biotin**-CGTGCCTGTTCTTCGCATCCAGCGCCGGGTTA	Attachment Biotin at 5'	L2
Bio-L3	**Biotin**-ATAATCAGAAAAGCCCAACATCCACTGTAATA	Attachment Biotin at 5'	L3
Bio-L4	**Biotin**-CATAGGTCTGAGAGACAAATCGTCGAATTACC	Attachment Biotin at 5'	L4

Supplementary Table 7.4. Modified staple strands given from the 5' to 3'end for the fluorescently-labeled L-shaped DNA origami structures. The complementary docking sequences are highlighted in orange. The docking strands have a three-base extension from the DNA origami structure and the pointer contains nine single-stranded thymine bases plus GC and the docking sequence, highlighted in grey.

ID	Sequence (5' to 3')	Supplier	Function	Replace
488-L8	TGC TCA TTC TXA TGC GTT AAT AAA ACG AAC TAT ATT CAT TGG CTT TTG; X = dT- Atto488	biomers	Lower Label – V1	L8
640-L9	AAG GGA ACC GYA TAT TCA CTC ATC TTT GAC CCG TAA TGC CAT CGG AAC; Y = dT- Atto647N	Eurofines Genomics	Upper Label – V1	L9
561-L7	GGCACCAAAACCAAAAGTAAGAGCAACACTATA GCAACGTAAATCGCCTTTTTTTTTCGGGCATTTA - Cy3b	Eurofines Genomics	Pointer - dye at 3'	L7
6.5nt-L6	GCACCCTCCGTCAGGTACGTTAGTAAATGAATAG TTAGCGTCAATCATTTTCAAATGC	Eurofines Genomics	Pos 12 catching site 7 nt *mismatch*	L6
7nt-L6	GCACCCTCCGTCAGGTACGTTAGTAAATGAATAG TTAGCGTCAATCATTTTTAAATGC	Eurofines Genomics	Pos 12 catching site 7 nt	L6
7.5nt-L6	GCACCCTCCGTCAGGTACGTTAGTAAATGAATAG TTAGCGTCAATCATTTTCAAATGCC	Eurofines Genomics	Pos 12 catching site 8 nt *mismatch*	L6
8nt-L6	GCACCCTCCGTCAGGTACGTTAGTAAATGAATAG TTAGCGTCAATCATTTTTAAATGCC	Eurofines Genomics	Pos 12 catching site 8 nt	L6
6.5nt-L5	AGAAACAGCTTTAGAAGGAAGAAAAATCTACGAT TTTAAGCATATAACTTTCAAATGC	Eurofines Genomics	Pos 6 catching site 7 nt *mismatch*	L5
7nt-L5	AGAAACAGCTTTAGAAGGAAGAAAAATCTACGAT TTTAAGCATATAACTTTTAAATGC	Eurofines Genomics	Pos 6 catching site 7 nt	L5
7.5nt-L5	AGAAACAGCTTTAGAAGGAAGAAAAATCTACGAT TTTAAGCATATAACTTTCAAATGCC	Eurofines Genomics	Pos 6 catching site 8 nt *mismatch*	L5
8nt-L5	AGAAACAGCTTTAGAAGGAAGAAAAATCTACGAT TTTAAGCATATAACTTTTAAATGCC	Eurofines Genomics	Pos 6 catching site 8 nt	L5
7.5nt-L14	TGCCATTCAACAATAGAAAATTCATATGGTTTTCA AATGCCTTTCAAATGCC	Eurofines Genomics	Pos 9 catching site 8 nt *mismatch*	L14
L12-13-I	CATTATACCAGTGATTTGGCATCAGGACGTTGTA ACATAAACCAGACG	Eurofines Genomics	Replacement for Pos 9	L12 and L13
L12-13-II	TAATAAGAAGAGCCACCCTTATTAGCGTT	Eurofines Genomics	Replacement for Pos 9	L12 and L13
L12-13-III	TTACCCATAACCCTCGAAATACAATGTTTAAACA GGG	Eurofines Genomics	Replacement for Pos 9	L12 and L13

SUPPLEMENTARY NOTE 8: STATISTICS SINGLE-MOLECULE DATA

The following section summarizes the results for all Hsp70 and origami datasets, newly designed, presented, and analyzed in this work. For each dataset, the total number (#) of recorded traces, the confidence interval, the number of dynamic traces, the retrieved number of states, and dwell times are specified. For the origami datasets, we refer to State 1 as the 12 o'clock position and State 2 as the 6 o'clock position for the 2-state systems. In 3-state systems (as presented in Figure 5), we refer to States 1, 2, and 3 as the 12 o'clock, 9 o'clock, and 6'clock positions, respectively. For the Hsp70 datasets, we refer to State 1 as the undocked conformation (low FRET) and State 2 as the docked conformation (high FRET). More details can be found together with the raw data on Zenodo [https://zenodo.org/record/7561162].

Supplementary Table 8.1. Statistics summarizing the single-molecule results obtained for the origami and Hsp70 datasets. N.A.: not applicable; max: highest confidence of all possible classes.

Fig.	Name	Binding Site	# of Traces	Confidence	Frame time (ms)	# of Frames	# of Dynamic Tracers	# of States	Dwell time t_1 (s)	Dwell time t_2 (s)	Dwell time t_3 (s)
2	1c origami 2 states	8 nt / 1 MM	7448	70 %	52.2	998	2510	2	1.75	2.65	N.A.
3	2c origami 2 states	8 nt / 1 MM	6100	70 %	52.2	1998	1499	2	1.76	2.64	N.A.
4	3c origami 2 states	7 nt / 1 MM	5731	20 %	34.2	2997	482	2	0.33	0.40	N.A.
		7 nt	5093	70 %	37.5	2997	1885	2	0.66	1.05	N.A.
		8 nt / 1 MM	2545	70 %	52.5	2397	581	2	1.75	2.69	N.A.
		8 nt	8097	70 %	152.2	1797	1545	2	6.41	9.54	N.A.
5	3c origami 3 states	7 nt @ 6 / 12h<br>8 nt / 1MM @ 9h	7990	70 %	52.5	2997	586	3	0.65	0.69	~1.40
6i	2c origami 2 states	5 nt	95	N.A.	N.A.	N.A.	95	2	0.0045	0.0063	N.A.
		6 nt	104	N.A.	N.A.	N.A.	104	2	0.015	0.026	N.A.
		7 nt / 1 MM	99	N.A.	N.A.	N.A.	99	2	0.14	0.23	N.A.
		7 nt	97	N.A.	N.A.	N.A.	97	2	0.84	1.62	N.A.
		6 nt ↔ 7 nt	11	N.A.	N.A.	N.A.	11	2	0.017	1.524	N.A.
		7 nt ↔ 7 nt / 1 MM	102	N.A.	N.A.	N.A.	102	2	0.79	0.22	N.A.
		7 nt / 1 MM ↔ 7 nt	21	N.A.	N.A.	N.A.	21	2	0.15	1.57	N.A.
**Fig.**	**Name**	**Glycerol concentration (%)**	**# of Traces**	**Confidence**	**Frame time (ms)**	**# of Frames**	**# of Dynamic Tracers**	**# of States**	**Dwell time t_1 (s)**	**Dwell time t_2 (s)**	**Dwell time t_3 (s)**
6a-b	3c origami 2 states 8 nt / 1 MM	1 %	1167	50 %	52.2	2397	176	2	1.65	2.45	N.A.
		2.5 %	1087	30 %	52.2	2397	150	2	1.52	2.23	N.A.
		5 %	1814	50 %	52.2	2397	382	2	1.38	2.02	N.A.
		10 %	1040	50 %	52.2	2397	120	2	1.11	1.52	N.A.
		15 %	1006	50 %	52.2	2397	204	2	0.96	1.23	N.A.
		20 %	1295	50 %	52.2	2397	101	2	0.85	1.11	N.A.
		30 %	1207	30 %	52.2	2397	101	2	0.62	0.85	N.A.
**Fig.**	**Name**	**ADP concentration (M)**	**# of Traces**	**Confidence**	**Frame time (ms)**	**# of Frames**	**# of Dynamic Tracers**	**# of States**	**Dwell time t_1 (s)**	**Dwell time t_2 (s)**	**Dwell time t_3 (s)**
6e-f	Hsp70 SSC1	100 nM	12319	max	32.5	2000	466	2	1.13	0.93	N.A
		5 µM	9246	max	32.5	2000	155	2	0.92	0.75	N.A
		100 µM	5035	max	32.5	2000	258	2	0.64	0.83	N.A
		1 mM	3534	max	32.5	2000	156	2	0.58	0.79	N.A

SUPPLEMENTARY REFERENCES

1 Abadi, M. *et al.* TensorFlow: Large-scale machine learning on heterogeneous systems. *ArXiv*, doi:10.48550/arXiv.1603.04467 (2015).

2 Tang, W. *et al.* Omni-Scale CNNs: a simple and effective kernel size configuration for time series classification. *ArXiv*, doi:10.48550/arXiv.2002.10061 (2022).

3 He, K., Zhang, X., Ren, S. & Sun, J. Deep Residual Learning for Image Recognition. *ArXiv*, doi:10.48550/arXiv.1512.03385 (2015).

4 He, K., Zhang, X., Ren, S. & Sun, J. Delving Deep into Rectifiers: Surpassing Human-Level Performance on ImageNet Classification. *2015 IEEE International Conference on Computer Vision (ICCV), Santiago, Chile*, 1026-1034, doi:10.1109/ICCV.2015.123 (2015).

5 Fukushima, K. Cognitron: a self-organizing multilayered neural network. *Biol. Cybern.* **20**, 121-136, doi:10.1007/BF00342633 (1975).

6 Smith, S. L., Kindermans, P.-J., Ying, C. & Le, Q. V. Don't Decay the Learning Rate, Increase the Batch Size. *ArXiv*, doi:10.48550/arXiv.1711.00489 (2018).

7 Thomsen, J. *et al.* DeepFRET, a software for rapid and automated single-molecule FRET data classification using deep learning. *eLife* **9**, e60404, doi:10.7554/eLife.60404 (2020).

8 Schreiber, J. Pomegranate: fast and flexible probabilistic modeling in python. *J. Mach. Learn. Res.* **18**, 1-6, doi:10.48550/arxiv.1711.00137 (2018).

9 Basden, A. G., Haniff, C. A. & Mackay, C. D. Photon counting strategies with low light level CCDs. *Mon. Not. R. Astron. Soc.* **345**, 985-991, doi:10.1046/j.1365-8711.2003.07020.x (2003).

10 Hirsch, M., Wareham, R. J., Martin-Fernandez, M. L., Hobson, M. P. & Rolfe, D. J. A Stochastic Model for Electron Multiplication Charge-Coupled Devices – From Theory to Practice. *PLoS ONE* **8**, e53671, doi:10.1371/journal.pone.0053671 (2013).

11 Murphy, K. P. *Probabilistic machine learning: an introduction*. (MIT press, 2022).

12 Messer, P. K., Henss, A. K., Lamb, D. C. & Wintterlin, J. A multiscale wavelet algorithm for atom tracking in STM movies. *New J. Phys.* **24**, 14, doi:10.1088/1367-2630/ac4ad5 (2022).

13 Wanninger, S. *et al.* Deep-Learning assisted, Single-molecule Imaging analysis of multi-color DNA Origami structures. *Zenodo*, doi:10.1101/2023.01.31.526220 (2023).

14 McKinney, S. A., Joo, C. & Ha, T. Analysis of Single-Molecule FRET Trajectories Using Hidden Markov Modeling. *Biophys. J.* **91**, 1941-1951, doi:10.1529/biophysj.106.082487 (2006).

15 Zarrabi, N., Schluesche, P., Meisterernst, M., Börsch, M. & Lamb, D. C. Analyzing the Dynamics of Single TBP-DNA-NC2 Complexes Using Hidden Markov Models. *Biophys. J.* **115**, 2310-2326, doi:10.1016/j.bpj.2018.11.015 (2018).

16 Sikor, M., Mapa, K., von Voithenberg, L. V., Mokranjac, D. & Lamb, D. C. Real-time observation of the conformational dynamics of mitochondrial Hsp70 by spFRET. *The EMBO Journal* **32**, 1639-1649, doi:10.1038/emboj.2013.89 (2013).

17 Krause, S. *et al.* Graphene-on-glass preparation and cleaning methods characterized by single-molecule DNA origami fluorescent probes and Raman spectroscopy. *ACS Nano* **15**, 6430-6438, doi:10.1021/acsnano.0c08383 (2021).

18 Kamińska, I. *et al.* Graphene Energy Transfer for Single-Molecule Biophysics, Biosensing, and Super-Resolution Microscopy. *Adv. Mater.* **33**, 2101099, doi:10.1002/adma.202101099 (2021).
