# Supplementary material for: Deep-LASI: deep-learning assisted, single-molecule imaging analysis of multi-color DNA origami structures
Source: Nat Commun. 2023 Oct 17;14:6564. doi: 10.1038/s41467-023-42272-9 (PMC10582187; doi:10.1038/s41467-023-42272-9)
Supplement: Supplementary file 3 — Reporting Summary [file 41467_2023_42272_MOESM3_ESM.pdf]

nature portfolio

Corresponding author(s): Evelyn Ploetz and Don C. Lamb

Last updated by author(s): 2023/09/04

Reporting Summary

Nature Portfolio wishes to improve the reproducibility of the work that we publish. This form provides structure for consistency and transparency in reporting. For further information on Nature Portfolio policies, see our Editorial Policies and the Editorial Policy Checklist.

Statistics

For all statistical analyses, confirm that the following items are present in the figure legend, table legend, main text, or Methods section.

n/a	Confirmed	
☐	☒	The exact sample size (*n*) for each experimental group/condition, given as a discrete number and unit of measurement
☒	☐	A statement on whether measurements were taken from distinct samples or whether the same sample was measured repeatedly
☒	☐	The statistical test(s) used AND whether they are one- or two-sided<br>*Only common tests should be described solely by name; describe more complex techniques in the Methods section.*
☒	☐	A description of all covariates tested
☒	☐	A description of any assumptions or corrections, such as tests of normality and adjustment for multiple comparisons
☐	☒	A full description of the statistical parameters including central tendency (e.g. means) or other basic estimates (e.g. regression coefficient) AND variation (e.g. standard deviation) or associated estimates of uncertainty (e.g. confidence intervals)
☒	☐	For null hypothesis testing, the test statistic (e.g. *F*, *t*, *r*) with confidence intervals, effect sizes, degrees of freedom and *P* value noted<br>*Give P values as exact values whenever suitable.*
☐	☒	For Bayesian analysis, information on the choice of priors and Markov chain Monte Carlo settings
☒	☐	For hierarchical and complex designs, identification of the appropriate level for tests and full reporting of outcomes
☒	☐	Estimates of effect sizes (e.g. Cohen's *d*, Pearson's *r*), indicating how they were calculated

Our web collection on statistics for biologists contains articles on many of the points above.

Software and code

Policy information about availability of computer code

Data collection	As described in the method section, synchronization and alternation of the excitating laser sources as well as the framewise data acquisition with up to 3 separate cameras was achieved using a LabView 2018-written programm that controls an field programmable gate array (FPGA). Upon starting the data acquisition, the FPGA synchronizes the execution (i.e. AOTF/LASER/Cameras) of the hardware via TTL pulses. The camera control is achieved using the supplier's software Andor Solis (Andor Technolgies, Belfast; UK), which finally records movies (image stacks) as standard tiff file per camera
Data analysis	Recorded tiff files were analyzed afterward by the new MATLAB-written program Deep-LAS,I which furthermore relies on the Python-based library "Pomegranate". The presented program Deep-LASI is published on GitLab [https://gitlab.com/simon71/deeplasi]. A description and documentation of the software can be found on ReadTheDocs at https://deep-lasi-tutorial.readthedocs.io/en/latest/index.html.

For manuscripts utilizing custom algorithms or software that are central to the research but not yet described in published literature, software must be made available to editors and reviewers. We strongly encourage code deposition in a community repository (e.g. GitHub). See the Nature Portfolio guidelines for submitting code & software for further information.

Data

Policy information about availability of data

All manuscripts must include a data availability statement. This statement should provide the following information, where applicable:

- Accession codes, unique identifiers, or web links for publicly available datasets
- A description of any restrictions on data availability
- For clinical datasets or third party data, please ensure that the statement adheres to our policy

The data for all figures and all supplementary figures have been deposited in the Zenodo database [https://zenodo.org/record/7561162], with the exception of previously published data (HSP70 SSC1 and DNA origami confocal data in Figure 6).

Human research participants

Policy information about studies involving human research participants and Sex and Gender in Research.

Reporting on sex and gender	not applicable.
Population characteristics	not applicable.
Recruitment	not applicable.
Ethics oversight	not applicable.

Note that full information on the approval of the study protocol must also be provided in the manuscript.

Field-specific reporting

Please select the one below that is the best fit for your research. If you are not sure, read the appropriate sections before making your selection.

☒ Life sciences ☐ Behavioural & social sciences ☐ Ecological, evolutionary & environmental sciences

For a reference copy of the document with all sections, see nature.com/documents/nr-reporting-summary-flat.pdf

Life sciences study design

All studies must disclose on these points even when the disclosure is negative.

Sample size	For training the neural network, we used simulated data set with ~ 200,000 traces as it is sufficient to cover an extensive range of realistic experimental parameters and thereby avoid any bias in the analysis. This includes FRET efficiencies between 0.01 and 0.99, dwell times of 1 to 100 frames and SNR of ~ 0.3 to 50. Experimentally, we typically measured 100 movies for each condition, as this typically generates several thousand acceptable traces.
Data exclusions	The full datasets were analyzed. The program, as part of its function, determines which intensity traces are suitable for further analysis. The computer selection was tested against simulated traces as well as compared with human analyses.
Replication	The Deep-LASI software was trained on three independently generated data sets. Deep-LASI was also compared with two users who manually analyzed the same data sets. All analyses reproduced the same results within acceptable tolerances.
Randomization	Randomization was not made since there is no advantage to randomizing the collected data, which have no specific ordering to begin with.
Blinding	The results expected from the experimental data were not known to the user. Hence, knowledge regarding the sample did not influence the manual selection or analysis of the data. For the neural network, the advantage is that it operates only based on the data that is has been trained with. Hence, no blinding is applicable here.

Reporting for specific materials, systems and methods

We require information from authors about some types of materials, experimental systems and methods used in many studies. Here, indicate whether each material, system or method listed is relevant to your study. If you are not sure if a list item applies to your research, read the appropriate section before selecting a response.

Materials & experimental systems

n/a	Involved in the study
☒	☐ Antibodies
☒	☐ Eukaryotic cell lines
☒	☐ Palaeontology and archaeology
☒	☐ Animals and other organisms
☒	☐ Clinical data
☒	☐ Dual use research of concern

Methods

n/a	Involved in the study
☒	☐ ChIP-seq
☒	☐ Flow cytometry
☒	☐ MRI-based neuroimaging
